# Transcranial Direct-Current Stimulation and Behavioral Training, a Promising Tool for a Tailor-Made Post-stroke Aphasia Rehabilitation: A Review

**DOI:** 10.3389/fnhum.2021.742136

**Published:** 2021-12-20

**Authors:** Marina Zettin, Caterina Bondesan, Giulia Nada, Matteo Varini, Danilo Dimitri

**Affiliations:** ^1^Centro Puzzle, Turin, Italy; ^2^Department of Psychology, University of Turin, Turin, Italy

**Keywords:** stroke, aphasia, post-stroke aphasia, transcranial direct current stimulation, neurorehabilitation, language training, tDCS

## Abstract

Aphasia is an acquired language disorder resulting from damage to portions of the brain which are responsible for language comprehension and formulation. This disorder can involve different levels of language processing with impairments in both oral and written comprehension and production. Over the last years, different rehabilitation and therapeutic interventions have been developed, especially non-invasive brain stimulation (NIBS) techniques. One of the most used NIBS techniques in aphasia rehabilitation is the Transcranial Direct-Current Stimulation (tDCS). It has been proven to be effective in promoting a successful recovery both in the short and the long term after a brain injury. The main strength of tDCS is its feasibility associated with relatively minor side effects, if safely and properly administered. TDCS requires two electrodes, an anode and a cathode, which are generally placed on the scalp. The electrode montage can be either unipolar or bipolar. The main aim of this review is to give an overview of the state of the art of tDCS for the treatment of aphasia. The studies described included patients with different types of language impairments, especially with non-fluent aphasia and in several cases anomia. The effects of tDCS are variable and depend on several factors, such as electrode size and montage, duration of the stimulation, current density and characteristics of the brain tissue underneath the electrodes. Generally, tDCS has led to promising results in rehabilitating patients with acquired aphasia, especially if combined with different language and communication therapies. The selection of the appropriate approach depends on the patients treated and their impaired language function. When used in combination with treatments such as Speech and Language Therapy, Constraint Induced Aphasia Therapy or Intensive Action Treatment, tDCS has generally promoted a better recovery of the impaired functions. In addition to these rehabilitation protocols, Action Observation Therapy, such as IMITAF, appeared to contribute to the reduction of post-stroke anomia. The potential of combining such techniques with tDCS would would therefore be a possibility for further improvement, also providing the clinician with a new action and intervention tool. The association of a tDCS protocol with a dedicated rehabilitation training would favor a generalized long-term improvement of the different components of language.

## Introduction

Aphasia is an acquired language disorder resulting from damage to the portions of the brain which are responsible for language comprehension and formulation. The most common causes of this disorder include vascular lesions, encephalic traumatic injury, and brain tumors (Marangolo and Caltagirone, 2014), with a prevalence of 250,000 cases in the United Kingdom and 1 million in the United States (Crinion, 2016). Aphasia may also be associated with other degenerative, inflammatory, autoimmune or parasitic disorders. About 0.7 to 3% of people presenting with multiple sclerosis also show aphasic symptoms (Naro et al., 2021).

Although damages to specific brain areas and their connections mainly occur in the left hemisphere, functional magnetic resonance imaging (fMRI) studies such as the one carried out by Thompson and den Ouden (2008) showed that in some cases the dominant language areas can be located in the right hemisphere.

Aphasia can involve different levels of language processing with impairments in both oral and written comprehension and production. Most patients who experience aphasia show some degree of spontaneous recovery within the first two to three months, due to a functional neural reactivation and reorganization. The most important factors that determine recovery are the lesion size and location, the type and severity of aphasia, the treatment received and, to some extent, the nature of early hemodynamic response (Watila and Balarabe, 2015).

From the second half of the twentieth century, different rehabilitation perspectives and therapeutic interventions for aphasia rehabilitation have been developed. The most recommended treatment for this disorder is Speech and Language Therapy (SLT). However, it is argued that SLT would lead to moderate effects, even when administered at high intensity. For this reason, over the last few years, new strategies have been implemented to enhance the effects of traditional rehabilitation.

These effects depend on the metaplasticity, which “refers to activity-dependent changes in neural functions that modulate subsequent synaptic plasticity such as long-term potentiation (LTP) and long-term depression (LTD)” (Abraham and Philpot, 2009). Changes in the pathomechanisms underlying psychiatric and neurological disorders are possible by acting on metaplasticity (Cantone et al., 2021). Non-Invasive Brain Stimulation Techniques (NIBS) can be a beneficial tool to transiently modulate cortical excitability and lead to lasting changes after the stimulation time (Fisicaro et al., 2020). Non-invasive brain stimulation techniques facilitate the activation of single brain areas, or the inhibition of other ones whose hyperactivation could have a maladaptive effect on cognitive recovery (Simonetta-Moreau, 2014). One of the main neuromodulation tools is Transcranial Magnetic Stimulation (TMS), which generates magnetic field pulses under the scalp. A single impulse leads to a short-term effect, while a sequence of stimulation on the same region of interest can generate long-term effects. These can either be inhibiting or excitatory, depending on the stimulation frequency (Fisicaro et al., 2020). For instance, repetitive TMS (rTMS) can successfully treat both motor and non-motor symptoms in stroke patients, including depression, which often affects the rehabilitation process after a stroke (Fisicaro et al., 2019). Both for safety and cost issues, tDCS is often preferred over TMS.

The main strength of tDCS is its feasibility associated with relatively minor side effects, if safely and properly administered. Another strength is that it shows promise as an effective and versatile neurostimulation tool. It has the potential to be a treatment for several conditions characterized by an alteration of the cerebral cortex activation. Indeed, it has been proven to have beneficial effects on both neuropsychiatric and neurological disorders, such as mood disorders, substance abuse, Alzheimer’s and Parkinson’s disease, multiple sclerosis, as well as post-stroke motor and cognitive impairment (Lefaucheur et al., 2017). Additionally, tDCS can be applied in sham mode, making it easier to carry out a single-blind study (Nitsche et al., 2003). Therefore, tDCS represents one of the most promising tools for the treatment of aphasia (Biou et al., 2019). It does not directly induce an action potential, but it delivers a continuous current flow at a low intensity (1/2 mA) instead. Transcranial Direct-Current Stimulation requires two electrodes, an anode and a cathode, which are generally placed on the scalp. Depending on the polarity and the consequent positioning of the electrodes, the experimenter can obtain a depolarizing effect, thus favoring neuronal firing (anodic tDCS), or a hyperpolarized effect by decreasing the discharge rate (cathodic tDCS) (Liebetanz et al., 2002). Because of the electrode size, tDCS allows the stimulation of large cortical areas, with a consequent reduction of stimulation focality. The effects of tDCS are variable and depend on the stimulation duration, the current density, the characteristics of the neuronal tissue involved and the current flow direction, which can move from the anode to the cathode or vice versa (Chase et al., 2020). Non-invasive brain stimulation is an important resource in neuropsychological rehabilitation, however, its application is not risk-free, as most non-invasive current induction tools. The ultimate goal of applying tDCS in rehabilitation is to re-establish an interhemispheric balance by promoting functional brain reorganization and facilitating relearning (Simonetta-Moreau, 2014).

To date, scientific literature offers a comprehensive overview of the several therapeutic treatments used for the rehabilitation of aphasia. Unfortunately, these many specific training methods lead to moderate effects. Therefore, a number of techniques have been implemented over the years in support of speech therapy and neuropsychological rehabilitation to promote a faster and more effective recovery. As tDCS is the most widely used method in rehabilitation, this review aims at examining those studies which associate rehabilitation with tDCS, and investigate its effectiveness (Marangolo, 2017).

## Summary of Findings

**FIGURE 1:**
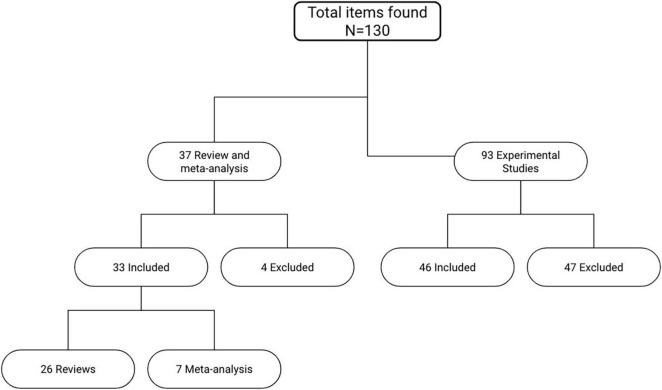
Flowchart of data gathering procedure.

Several articles were selected and analyzed for this review. PubMed, PsycInfo and Cochrane were consulted for the systematic search of the relevant articles. As for keywords, different combinations of the terms *“aphasia,” “speech impairment,” “Broca’s aphasia,” “non-fluent aphasia,” “tDCS,” “transcranial direct current stimulation,” “non-invasive brain stimulation,” “cognitive rehabilitation,” “neurorehabilitation,” “aphasia training,” “cognitive training,” “language recovery”* were used. The whole research process started from an accurate analysis of the most quoted and detailed reviews available on this topic. Out of the 37 most relevant reviews and meta-analysis, only 33 specifically analyzed tDCS studies on aphasic patients with acquired cerebral lesions (Figure 1). The experimental studies were subsequently extracted from these reviews. From a total of 93 studies analyzed, 46 were considered the most relevant. The main exclusion criterion was the type of aphasia: only tDCS studies conducted on a sample of aphasic patients at the chronic phase with an acquired cerebral lesion were included. Studies on other types of aphasia (e.g., primary progressive aphasia) or carried out earlier than six months from the damage were excluded. Moreover, it was decided to include only studies enrolling a minimum of three subjects, thus removing single-case studies from the total count of papers. The conclusions drawn from those studies could have been weak and not significant enough for the purpose of this study. All experimental studies using TMS or other brain stimulation techniques other than tDCS were not included as well. Out of the 79 articles included in this review, 26 were reviews, 7 meta-analysis and 46 were experimental studies (Supplementary Table 1).

## Patients

The studies here described included patients with different types of language impairments. Patients presented with both fluent and non-fluent aphasia, and in several cases anomia (Vines et al., 2009, 2011; Flöel et al., 2011; Fridriksson et al., 2011, 2018b; Richardson et al., 2015; Basat et al., 2016) or a deficit in spontaneous speech production (Marangolo et al., 2014a; Guillouët et al., 2020). Apraxia of speech was also often found (Marangolo et al., 2011, 2013a; De Aguiar et al., 2015a).

In most research, with symptoms lasting at least six months after stroke, aphasia was regarded as chronic. Although some studies also enrolled patients with subacute damage, these were not included in the final references. It is in fact suggested that tDCS targeting perilesional areas in the acute or subacute phase after stroke could lead to limited language improvements (Zeiler, 2019). In the first months after the onset, most patients can in fact already exhibit a partial spontaneous recovery due to specific neural mechanisms (Zeiler, 2019). This first spontaneous recovery often involves some areas of the healthy hemisphere (Saur et al., 2006; De Aguiar et al., 2015a). This occurrence is in line with the theory of interhemispheric inhibition (Liepert et al., 2000; Pascual-Leone et al., 2005), which claims that in the intact human brain, each hemisphere can inhibit the other one to keep an interhemispheric balance and to prevent an excessive interference between the activity of both hemispheres (Pascual-Leone et al., 2005). In the event of a brain damage, the ability of the left hemisphere to inhibit the right one is limited, and this could bring to an increase of the excitability of the healthy hemisphere, together with an increase of the inhibitory signal toward the damaged one (Saur et al., 2006). Only at a later stage of recovery the healthy hemisphere would start sending excitatory signals toward the damaged areas, allowing the perilesional tissue to reactivate in a first attempt to restore the impaired functions (Pascual-Leone et al., 2005). These spontaneous dynamics occur in the first six months after injury, hence it is advisable the application of tDCS in chronic aphasic post-stroke patients rather than in the acute or subacute phase. At a chronic stage, it would also be easier to understand which neural connections are preserved and which areas are more active during the execution of specific cognitive tasks (Lytton et al., 1999). Additionally, the perilesional activity seems to be stronger and more stable after six months (Cassidy and Cramer, 2017). For these reasons, tDCS applied to perilesional areas on subjects with chronic aphasia would bring to a better recovery (De Aguiar et al., 2015b).

However, there is also some evidence to suggest that patients who managed to achieve a full language recovery and exclusively showed an activity in the homologous language areas of the right hemisphere (Thompson and den Ouden, 2008; Marini et al., 2016). It is believed that this has a greater chance of occurring in case of more severe left hemisphere damage leaving little to no perilesional tissue left (Marini et al., 2016). In these cases, the inhibitory signal toward the healthy hemisphere would be missing and the right hemisphere would consequently be hyperactive (Thompson and den Ouden, 2008). In case of more severe damages, right anodal tDCS is in fact recommended rather than cathodal montage. It must also be remembered that language processing does not exclusively occur in the left hemisphere. Hence, using fMRI to locate the areas that are activated during specific tasks is generally suggested before running tDCS trials (Marshall et al., 2000). In this way, it would be easier to understand which connections could be strengthened through tDCS. In chronic aphasia, the neural connections stabilize after the first period of spontaneous recovery. Performing a fMRI scan on a subject with chronic aphasia would therefore guarantee a better picture of what the spared language areas are. fMRI would allow a tailor-made electrode montage with the purpose of enhancing the spared connections specifically for the subject treated (Marshall et al., 2000). Several studies relied on fMRI to detect the most active perilesional areas, in order to set up a personalized perilesional montage (Baker et al., 2010; Rosso et al., 2014; De Aguiar et al., 2015a; Richardson et al., 2015; Darkow et al., 2017). These studies showed significant improvements in patients’ conditions.

Although considered safe, tDCS requires a specific list of exclusion criteria in order to remove high-risk patients from the trials. Primarily, subjects with a history of epilepsy, psychiatric or neurological conditions must be excluded. Drug consumption and the use of medications are not considered safe if combined with tDCS. Additionally, most tDCS studies also exclude people with brain tumors from the sample. These generally have a slow and gradual growth, which could already lead to a brain reorganization before an eventual brain damage. Thus, the reorganization of neural networks could follow a different path compared to that pertaining to patients without brain tumors (Schlaug et al., 2011). Enrolling patients and reaching a significant sample size can therefore be a long process. Hence, amongst the studies here analyzed, the average sample size is 16/17 participants, with studies going from a minimum of 3 (Fiori et al., 2011; Marangolo et al., 2011; Vestito et al., 2014) to a maximum of 74 patients enrolled (Cramer, 2018; Fridriksson et al., 2018a,b).

## Mechanisms Underlying a Left Hemisphere Damage

After a left hemisphere injury, two neural processes can occur. In some cases, the spared tissue surrounding the lesion can help recover the compromised functions, while in other cases the homologous areas of the right hemisphere are responsible for the recovery (Hamilton et al., 2011). To date, numerous studies suggest that the activation of the healthy hemisphere most likely occurs in the first months after stroke, and does not lead to a satisfactory recovery of the impaired functions. It is also suggested that the healthy hemisphere would intervene in case of a greater loss of cerebral tissue. Conversely, a perilesional neural activation would often occur in case of a localized and less severe injury, and would contribute to a better recovery (Schlaug et al., 2011; AlHarbi et al., 2017). After a brain injury, the interhemispheric balance is compromised. In the healthy brain, all functions work in harmony because of the constant interhemispheric competition. This phenomenon refers to the continuous inhibitory control between the two hemispheres, mediated by transcallosal connections: an increase in the activity of one of the hemispheres is therefore associated to a stronger inhibition toward the homologous areas of the opposite hemisphere (Bütefisch et al., 2008). The main goal of the interhemispheric competition process is to avoid excessive neural noise, which could disrupt the execution of cognitive tasks. Thus, in case of an injury in the left hemisphere, this part of the brain would decrease its activity and reduce its inhibitory signal toward the right one. However, the healthy hemisphere could still inhibit the impaired one, leading to its further hypoactivation and to a stronger imbalance (Murase et al., 2004). The main goal of most tDCS studies on aphasia is to prevent this imbalance, by potentiating the healthy neural connections and safeguarding the patients from a further decline of their impaired domains.

The role of the right hemisphere in language recovery is still unclear and debated, and for years it had been considered as dysfunctional. However, several studies underlined the potential benefits of its activation after an injury of the left hemisphere (Cheng et al., 2021). Starting from the nineteenth century, Barlow (1877) first described the case of a ten-year-old child who managed to recover his linguistic functions after a stroke; however, he lost them again after a second injury located in the right hemisphere. Further studies also showed that several subjects who underwent a left hemispherectomy were then able to restore their linguistic abilities, despite the removal of the left hemisphere, dominant for the processing of language functions (Vargha-Khadem et al., 1997). This would demonstrate the great vicarious power of the brain and its ability to restore its functions even after a significant loss of tissue.

Further evidence about the involvement of the right hemisphere after a left stroke is provided by studies that combined fMRI with language tasks. For instance, while undergoing a fMRI scan, a group of patients who suffered from a stroke in their left hemisphere showed both an activation of their left frontotemporal regions and an activity of the right homologous areas (Basso et al., 1989; Buckner et al., 1996; Gold and Kertesz, 2000). However, these fMRI studies do not provide a flawless explanation of the causal role between language recovery and the activation of the right hemisphere (Schlaug et al., 2011). This hypothesis was further investigated by implementing different NIBS techniques. Various studies using right anodal tDCS in conjunction with treatments based on Melodic Intonation Therapies (MIT) have in fact shown an improvement of verbal fluency in a group of aphasic participants whose lesions had severely affected their left hemisphere (Vines et al., 2009, 2011). Thus, it is clear that the right hemisphere could play a crucial role in language recovery after an acquired brain damage. The remaining open question deriving from these findings is in what circumstances would a right hemisphere intervention be adaptive and advantageous to aphasic patients (Crosson et al., 2007). Its benefit could in fact depend on several factors, such as size and severity of the damage. The right hemisphere is thought to play a bigger role in case of larger tissue loss in the left one (Heiss and Thiel, 2006). However, it is also important to know how lateralized the subject’s language functions were before the injury (Hamilton et al., 2011). The involvement of the right hemisphere would also vary depending on the stage after onset: different patterns of cerebral activity can be shown at different stages in the recovery process (Saur et al., 2006). It is believed that after a few weeks from the onset, the initial language improvements would be associated with a stronger activity in the right inferior frontal gyrus, the insula and the right supplementary motor area. After three months, the right perilesional areas would then play a bigger role in the recovery (Saur et al., 2006). The right hemisphere could therefore have a facilitatory and adaptive role in the acute and subacute phases, but be maladaptive at a chronic stage, since it would prevent the perilesional spared tissue from activating and contributing to the major recovery (Heiss and Thiel, 2006).

## tDCS-Induced Synaptic Plasticity

Generally, anodal stimulation (A-tDCS) facilitates the depolarization of the membrane potential and increases neuronal firing and cortical excitability. Cathodal stimulation (C-tDCS) mostly leads to the hyperpolarization of the membrane potential, thus decreasing neuronal firing and cortical excitability (Nitsche et al., 2003).

Although changes in the membrane potential are transitory, tDCS can help strengthen the synaptic connections by producing long-lasting effects that persist after the cessation of stimulation (Stagg and Nitsche, 2011). A prolonged stimulation can in fact result in long-term potentiation (LTP). Hebb (2005) described LTP as the strengthening of the neural connection between two neurons that fire simultaneously. Likewise, long term depression (LTD) refers to a lasting decrease in neuronal firing. These two phenomena represent a strengthening and a weakening of synaptic connections, respectively, essential for the acquisition and preservation of new information.

Both neurotransmitters and neuromodulators determine plasticity and tDCS can mediate their neuroregulation (Caumo et al., 2012). For instance, anodal tDCS on the primary motor cortex of healthy subjects generates a reduction in GABA concentration, whereas a cathodal stimulation results in a decrease of glutamate levels and consequently of GABA, as this is synthesized by glutamate (Stagg et al., 2009).

Further authors highlighted the role of nitric oxide (NO) as a new mediator of the effects of tDCS in promoting long-term potentiation (Barbati et al., 2020). N-methyl-D-aspartate (NMDA) receptors also appear to be involved in tDCS-induced synaptic plasticity. Nitsche et al. (2003) observed that by administering a NMDA receptor antagonist, tDCS seemed to have no effects.

Additionally, serotonin and dopamine appear to play a key role, facilitating excitatory and inhibitory stimulation, respectively. However, their interaction with tDCS can be unclear and new studies are needed to better understand their role (Sandars et al., 2016). Another explanation of how tDCS can elicit long-term changes in the brain has been given by Ardolino et al. (2005), who hypothesized that such changes might result from stimulation-induced non-synaptic mechanisms. According to their study, axonal molecules could change their conformation and function when exposed to direct current stimulation.

Plastic changes are also observed with different neuromodulation techniques, such as low-frequency Repetitive Transcranial Magnetic Stimulation (1-Hz rTMS) and Non-invasive High Frequency Repetitive Transcranial Magnetic Stimulation (HF-rTMS). In a study carried out by Cambiaghi et al. (2021), the authors investigated the effects of these non-invasive techniques after a stimulation of the primary motor cortex of mice, thus observing an increase in the length of the dendritic spines. The results of these stimulation techniques relate to changes in dendritic complexity in the primary motor cortex that can strengthen corticocortical connections by increasing the integration of information across cortical areas (Cambiaghi et al., 2020, 2021).

## Electrode Montage

**FIGURE 2:**
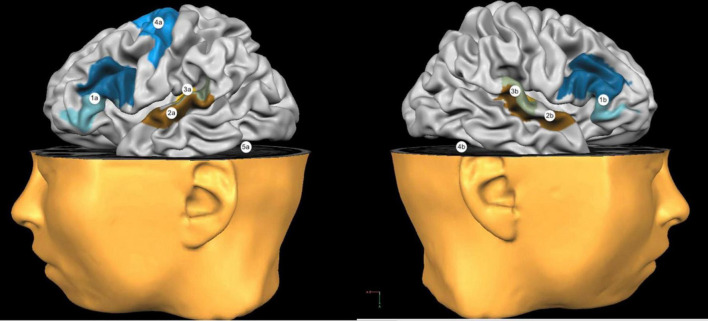
Most used stimulation sites. 1a: Broca’s Area; 2a: Wernicke’s Area; 3a: Left Temporoparietal Cortex; 4a: Left Primary Motor Cortex; 5a: Left Cerebellar Hemisphere. 1b: Right Broca’s Homologous Areas; 2b: Right Wernicke’s Homologous Areas; 3b: Right Temporoparietal Cortex; 4b: Right Cerebellar Hemisphere.

The electrode montage of tDCS can be either unipolar or bipolar. In the first case, one electrode is positioned on the scalp above the region of interest, while the reference is placed on an extracephalic area, such as the deltoid muscle. A bipolar montage, on the other hand, requires the positioning of both electrodes on the scalp. Generally, in case of bi-hemispheric stimulation studies, the anode is placed on one hemisphere and the cathode on the homolog areas of the opposite hemisphere. If only one active electrode is needed, this would be put above the interested cerebral area, while the reference would be placed on the supraorbital area of the opposite hemisphere (Lefaucheur et al., 2017). The most frequently used stimulation sites are portrayed in Figure 2.

As for the positioning of the reference, most studies using a bipolar montage set it on the supraorbital area of the hemisphere opposite to the stimulated one (Rosso et al., 2014; Meinzer et al., 2016; Branscheidt et al., 2017; Darkow et al., 2017; Fridriksson et al., 2018a,b; Pestalozzi et al., 2018; Spielmann et al., 2018; Woodhead et al., 2018). Other studies chose an extracephalic montage instead, by locating the reference on the deltoid, contralaterally to the active electrode (Baker et al., 2010; Shah-Basak et al., 2015; Marangolo et al., 2017; Norise et al., 2017; Zhao et al., 2021). An extracephalic reference could, in fact, minimize the chance of inducing an involuntary current flow underneath the electrode, which could lead to confounding effects about the influence of tDCS on the eventual recovery (Baker et al., 2010). On the other hand, the electric field force is thought to get weaker when the electrodes are more distant (Biou et al., 2019). Hence, placing the reference on an extracephalic spot (i.e., the deltoid) would lead to a weaker current propagation underneath the active electrode, and the beneficial role of tDCS could be less than expected (Nitsche et al., 2007). To overcome this occurrence, several studies located both electrodes on the scalp and increased the reference size. By doing this, the current density propagated by the inactive electrode would get lower, therefore reducing the risk of generating an unintentional current flow below it (Nitsche et al., 2007).

## Polarity and Site of Stimulation

In line with the interhemispheric competition model, most tDCS studies on aphasic patients opted for anodal stimulation on the spared areas of the left hemisphere in order to enhance its cortical perilesional activity (Marangolo, 2017). Several studies also used cathodal stimulation on the homologous language areas of the right hemisphere to reduce the excitability of those areas and to avoid an excessive inhibition of the spared perilesional tissue (Fiori et al., 2019). Bi-hemispheric stimulation with left anodal and right cathodal stimulation was also often used (Lee et al., 2013).

As for anodal stimulation, Baker et al. (2010) reached significant results after administering online perilesional A-tDCS combined with a picture-matching task. The treatment lasted for five consecutive days, in daily sessions of 20 min each. The main improvement in the subjects’ performance was related to the accuracy in object naming, and the progress persisted at the follow-up, two weeks after the end of the sessions. Other studies also obtained significant results by combining left frontal tDCS and language tasks. Generally, subjects showed major progress in articulation (Marangolo et al., 2011), naming accuracy (Vestito et al., 2014), picture description and sentence building (Campana et al., 2015). A-tDCS administered on Wernicke’s area showed an improvement on naming accuracy and speed, too (Fiori et al., 2011). Pestalozzi et al. (2018) specifically stimulated the left dorsolateral prefrontal cortex (DLPFC) with A-tDCS, while asking for naming and repetition tasks, and obtained a general improvement in verbal fluency and naming of high-frequency words. Few studies also chose to apply A-tDCS on areas not directly connected to language, such as the primary motor cortex (M1) (Meinzer et al., 2016; Branscheidt et al., 2017; Darkow et al., 2017) or the tenth thoracic vertebra through tsDCS (Marangolo et al., 2017). In the latter case, no significant differences were detected between the experimental conditions. It is, however, believed that spinal stimulation might contribute to improvements on action naming (Marangolo et al., 2017).

Nevertheless, several authors debated that in some cases a right hemisphere anodal stimulation might be beneficial at a chronic stage (Vines et al., 2009, 2011). In their studies, right frontal anodal tDCS, combined with MIT (Albert et al., 1973), brought to a noteworthy improvement in verbal fluency in the aphasic participants. Melodic intonation therapies is thought to employ right frontal areas to facilitate speech, hence right anodal tDCS might be advantageous if paired with melodic therapies (Vines et al., 2011).

In contrast, C-tDCS studies mainly focused on the inhibition of the homologous of Broca’s area on the right hemisphere. The most relevant ones are those carried out by Kang et al. (2007, 2011), Jung et al. (2011), Rosso et al. (2014), and Fiori et al. (2019). Although significant, Jung et al. (2011) did not provide a control condition, making the correlation between tDCS and linguistic improvement unclear. In a similar study, Kang et al. (2011) provided a sham-controlled condition but did not show a significant difference between sham and active tDCS. Rosso et al. (2014) argued that right C-tDCS could have different effects depending on the size and location of the lesion. In their study, patients with a focal lesion of Broca’s area benefited from a right cathodal stimulation, while those with lesions adjacent to Broca’s area did not show a significant improvement.

A bi-hemispheric montage was chosen by less researchers (Lee et al., 2013; Marangolo et al., 2013a, 2014b, 2016; De Aguiar et al., 2015a; Guillouët et al., 2020; Pisano et al., 2021), but often showed a significant performance improvement after a simultaneous right cathodal inhibition and left anodal stimulation, when paired with naming and reading tasks (Lee et al., 2013; Marangolo et al., 2013a, 2014a, 2016). A recent study tested the patients’ spontaneous speech after frontal bi-hemispheric tDCS, but the authors could not report a significant difference in the performances of the experimental group, when compared to the sham group (Guillouët et al., 2020). With bi-hemispheric tDCS it can be difficult to determine whether an eventual improvement is primarily caused by the stimulation of left perilesional tissue or by the inhibition of the right hemisphere (De Aguiar et al., 2015b). Caution in drawing causal inferences from these studies is therefore needed.

## Stimulation Parameters

The effects of tDCS are variable and depend on several factors, such as electrode montage and size, duration of the stimulation, current density and characteristics of the brain tissue underneath the electrodes. In all the studies analyzed, the electrodes area always varied from 25 cm^2^ to 35-36 cm^2^. It is generally claimed that the active electrode reduction would overcome tDCS biggest limit, namely its low spatial resolution.

All studies used a stimulation intensity of either 1 mA (Baker et al., 2010; Fridriksson, 2011; Rosso et al., 2014; Meinzer et al., 2016; Cherney et al., 2021) or 2 mA (Kang et al., 2011; Saidmanesh et al., 2012; Campana et al., 2015; Marangolo et al., 2018; Woodhead et al., 2018; Guillouët et al., 2020). Few studies, however, chose an intensity of 1.2 mA (Vines et al., 2009, 2011) or 1.5 mA (Vestito et al., 2014).

Opting for higher intensities, such as 2 mA, might lead to a stronger stimulation and guarantee a better outcome. However, a stimulation higher than 2 mA is generally not recommended for different reasons (De Aguiar et al., 2015b). Firstly, higher stimulations might influence the neural activity of the brain tissue adjacent to the region of interest (De Aguiar et al., 2015b). In terms of safety, intensities higher than 2 mA could burn and itch the scalp, thus they are not recommended (De Aguiar et al., 2015b). Additionally, a high stimulation intensity might make the subject perceive a feeling of discomfort while undergoing an active-tDCS experimental condition. Hence, a higher amperage can critically prejudice a sham controlled experimental study (O’Connell et al., 2012). Batsikadze et al. (2013) also showed that a 15-min 2 mA cathodal stimulation administered on the primary motor cortex increased the patients’ cortical excitability instead of decreasing it. A subsequent experimental session run at 1 mA, seemed to correctly inhibit the interested areas, as expected. It was therefore argued that higher intensities might cause opposite effects to those predicted (Batsikadze et al., 2013).

In all studies analyzed, tDCS was never used for more than 30 min. On average, 20-min-session stimulations seemed preferable (De Aguiar et al., 2015b). Protracted sessions might in fact lead to excessive cortical excitability, which would worsen the neurons’ activity in the long term, instead of improving it (De Aguiar et al., 2015b).

The number of tDCS sessions generally varied from 1 to 30. A review carried out by Rosso et al. (2018) indicated that repeated sessions can lead to greater improvements, if compared with studies that required less tDCS sessions. The authors claimed that greater language improvements were shown in those studies that used at least five tDCS sessions. Long-term improvements could be in fact attributable to the short-term effects of multiple sessions of transcranial stimulation, which might lead to a significant result (Holland and Crinion, 2012).

It is therefore suggested that the effects of tDCS might be dose-dependent (Rosso et al., 2018). However, it is necessary to guarantee a sufficient time interval between sessions: two subsequent sessions with a shorter interval in between might, in fact, lead to opposite effects than those expected. For instance, Fricke et al. (2011) revealed an inhibitory effect following two subsequent anodal stimulations, and attributed this event to the inadequate interval between sessions (only 3 min). As expected, a following stimulation attempt with a 30-min-interval between sessions showed an excitatory effect instead (Fricke et al., 2011).

Generally, it can be concluded that repeated tDCS sessions of 20 min each are advised, the intensity required should not differ from 1 to 2 mA, and the active electrodes should preferably have an area of 25 cm^2^.

## Aphasia Treatments

Choosing the right aphasia treatment and predicting its possible outcome depend on several variables. According to the location of the injury, different approaches can be chosen. Chapey (2008) identified three main categories of rehabilitation approaches, i.e., traditional, cognitive and specialized (Chapey, 2008).

Amongst the traditional treatments, one of the most important is the *Schuell’s Stimulation Approach* (Schuell et al., 1964). This utilizes repetitive, controlled language auditory stimuli to maximize the patients’ recovery. Schuell et al. (1964) reported the efficacy of this traditional technique, with the exception of patients with a severely impairing irreversible aphasia. Two other common traditional exercises are those pertaining to naming and picture description, such as the Picture Naming Task (PNT) and the Picture Description Task (PDT) (Galletta et al., 2016). Another widely used traditional treatment is the Thematic Language Stimulation (TLS), a systematic method that requires the practitioner to choose vocabulary items and use them as stimuli in different contexts (i.e., in different sentences or in response to a question). The goal of TLS is to facilitate better language processing, thereby making it possible for the patient to reach a more functional communication (Schuell et al., 1964; Wepman, 1972).

Cognitive approaches, on the other hand, solely focus on the patient’s impaired language domain. Thus, they require a precise identification of the levels affected. In this case, the rehabilitation process is specifically directed toward the recovery of one or few targeted functions. When a satisfactory recovery of the functions is not feasible, compensatory communication strategies can be thought of instead (Byng and Coltheart, 1986). Psycholinguistic treatments, such as the Language Oriented Treatment (LOT) (Helm-Estabrooks and Albert, 1991) can also be considered as belonging to the cognitive approach category. LOT is designed to provide a highly individualized and tailored approach to treatment based on the language profile. LOT major components are: stimulus, response, and reinforcement. In contrast to operant conditioning, however, the goal is not to learn specific stimulus–response connections, but to present stimuli followed by responses with feedback enabling patients to use language at appropriate levels. To improve the patients’ response, the complexity of the stimuli chosen and the demands made can vary according to the individual’s condition and their spared language abilities (Helm-Estabrooks and Albert, 1991).

Conversely, specialized treatments are usually addressed to moderate-to-severe aphasia types. In most cases, treatments are aimed at the compensation of the lost functions, for example through specific strategies or the use of electronic devices that can help to communicate. Augmentative and Alternative Communication (AAC) (Beukelman, 1985) is a specialized multimodal treatment addressed to people with severe speech and hearing impairments. The main goal of AAC is to guarantee an easy and accurate way to comprehend what an interlocutor says (Garrett and Beukelman, 1998). Thus, the primary attention is directed toward the receptive aspects of communication rather than the expressive ones. The use of drawing, writing of keywords, gestures, environmental contexts and prosody are common compensatory strategies to help reach a satisfactory level of communication with the patient (Garrett and Beukelman, 1998). Indeed, Conversational Therapy (CT) (Simmons-Mackie et al., 2000) identifies aphasia as a communication impairment. According to CT, in case of non-fluent aphasia, spontaneous speech production in an ecological setting might lead to a better use of communication skills, therefore helping communicate with third parties. In CT, the main goal of the therapist is to start a conversation with the aphasic patient, where both are actively involved in the conversation (Basso, 2003). Both the patient and the therapist are free to use every means to communicate at best, and optimally exchange relevant information, which includes the use of gestures, drawings, body language, phonological or orthographic cues. The therapist is taught to accept all information the patient is able to provide, and give them a meaning inside the context (Galletta et al., 2016). Another specialized treatment is the Melodic Intonation Therapy (MIT) (Sparks, 2008), which is generally beneficial in case of non-fluent aphasia types with limited outputs. Specifically, it uses melody and prosody to facilitate the patient’s language production (Galletta et al., 2016).

Speech and Language Therapy (SLT) and the Constraint Induced Aphasia Therapy (CIAT) are amongst the most widely used approaches (Chapey, 2008). The former defines a rehabilitative approach that can include various exercises, chosen by the therapist in line with the patient’s needs and abilities. The latter, on the other hand, aims to minimize non-verbal communication and discourage the use of compensatory strategies, to solely focus on verbal production (Shah-Basak et al., 2016; Norise et al., 2017). The main idea behind CIAT is that patients with acquired aphasia might be inclined to use compensatory strategies to avoid using their residual language skills. Gestures, for instance, are broadly overused. CIAT focuses on oral communication and forbids any other communication channel and it is one of the most used group therapies (Berube and Hillis, 2019).

Another common choice for group therapies is the Intensive Action Treatment (IAT), which specifically works on action observation and imitation. Its aim is to trigger both a frontal and a parietal activity, and specifically the areas attributable to the mirror neurons system. Observation with Intent To Imitate (OTI) and Action Observation Therapy (AOT) are two frequently used intensive treatments (Chapey, 2008). Over the past 30 years, the systemic use of computers and different softwares in the neurorehabilitation fields has allowed an expansion of rehabilitative techniques, thus facilitating both therapists and patients. As for conventional therapies, the efficacy of rehabilitation in face-to-face settings depends on numerous variables, both linguistic and non-linguistic, also related to the therapy setting (Hillis, 1998). Conversely, with computer-based individualized neuro-linguistic therapies it is easier to control the variables relative to the setting (Van de Sandt-Koenderman, 2011).

Three computer-based treatments, amongst others, significantly help aphasic patients to improve their communication skills. The first one is the Computer-Only Treatment (COT), which allows the patients to practice on their own, without the supervision of a therapist. Progresses and results can be checked later with the therapist. For example, a self-administered computerized treatment consisting of a spoken word–picture matching task can be applied (Fridriksson et al., 2011). Instead, Computer-Assisted Treatment (CAT) requires the presence of a supervisor while presenting all tasks on the computer. Lastly, computers can be used as an *electronic pointing device* to share text, images, digital animations and speech files between therapist and patient, so that both parties can communicate together during therapy sessions (Chapey, 2008). A significant improvement after the use of computer-based therapies was often shown (Fridriksson et al., 2012, 2018b; Richardson et al., 2015; Meinzer et al., 2016) and progresses were often maintained at the follow-up, which varied from two weeks (Fridriksson et al., 2011; Ihara et al., 2020) to three months (Woodhead et al., 2018).

Normally, aphasia rehabilitation requires a face-to-face setting, while telerehabilitation adopts a different paradigm. The employment of digital tools gives the patient the chance to perform rehabilitative tasks autonomously. In addition, the use of devices in rehabilitation increases motivation, self-esteem and adherence to treatment (Maresca et al., 2019).

Thanks to great technological progress, it is now possible to start therapies – also called *e-therapies* – remotely, relying on computers (Doogan et al., 2018; Maresca et al., 2019). E-therapies are helpful, especially for patients with physical disabilities or a limited access to rehabilitation centers and are therefore considered a great resource. Additionally, they allow more people to benefit from an accessible form of rehabilitation and they are thought to increase the compliance to therapy (Brennan et al., 2004; Theodoros et al., 2008; Laver et al., 2017). Among the aphasia rehabilitation protocols adopting computerized tasks we have identified *Power-Afa*, an Italian software composed of phonological, semantic, orthographic, morphological and syntactic tasks of increasing complexity (De Luca et al., 2018) and *iReadMore*, which uses a cross-modal and lexical approach. The latter consists in pairing written, spoken and images of words over multiple conditions, with increasing levels of difficulty. The program aims at strengthening language production, by improving the connections between the impaired language domains involved (Woodhead et al., 2018). As a result of studies using a mixed treatment approach, both individual and group therapy have been found to be effective in improving linguistic outcomes (Berube and Hillis, 2019).

## Aphasia, Transcranial Direct-Current Stimulation and Treatment Selection

Over the last years, non-invasive brain stimulation techniques, combined with different language and communication therapies, have often led to promising results in rehabilitating aphasic patients (Marangolo et al., 2017; Biou et al., 2019).

Transcranial Direct-Current Stimulation (tDCS) can be administered both online or offline. Online tDCS is based around the idea that cortical activity might increase if the electrical stimulation is paired to specific language tasks. Offline stimulation, on the other hand, might facilitate neuronal boost of the stimulated areas, which can later help in the execution of language tasks in a consequent treatment (De Aguiar et al., 2015b).

A study carried out by Marangolo (2013) showed that administering picture naming tasks after A-tDCS on Broca’s area led to an improvement of the patients’ naming skills. However, this did not happen if the anode was placed on Wernicke’s area. This shows that a more satisfactory result can be reached when the most appropriate electrical stimulation task is chosen. This highlights the codependent relation between task-dependent effects and stimulation site (Jacquemot et al., 2012). The effects of tDCS can also depend on the signal-to-noise ratio in the stimulated brain network. The use of A-tDCS during easier tasks yields a high signal-noise ratio, since the tasks might be more likely to involve an already consolidated neural network. In this case, A-tDCS would mostly cause firing in task-relevant neural areas. With practice, the signal-noise ratio would increase, leading to performance improvements. Conversely, in a more difficult task, the level of noise would be higher. In this case, A-tDCS might increase both noise and signal to a similar extent, thus hindering a facilitative effect. A decrease in firing rates due to C-tDCS would also lead to task-dependent behavioral consequences. With easier tasks, no particular benefit would result from decreasing general noise. However, with more complex tasks, C-tDCS may filter irrelevant activations, hence increasing the signal-noise ratio, resulting in performance facilitation (Miniussi et al., 2013).

Most tDCS studies on patients with acquired aphasia showed significant results. However, results are generally heterogeneous due to a number of variables such as sample size, aphasia type, participants’ age, lesion location and size and, in few cases, socio-cultural factors (Ulanov et al., 2019). Such heterogeneity is likely also due to the lack of a physiological assessment of patients, especially with regards to acute stress and post-stroke depression. Stroke patients are known to be at risk of developing anxiety, depression, negative thinking and post-traumatic stress disorders (Loubinoux et al., 2012), which are thought to impair the brain homeostasis and lead to a maladaptive plasticity. Several studies showed how neuromodulation and brain plasticity can be affected by underlying mental disorders (Kim and Diamond, 2002; Citri and Malenka, 2008; Khazen et al., 2018). According to a study carried out by Infortuna et al. (2021) on a healthy group of participants, changes can be seen in the motor cortex neuroplasticity after a stress-inducing task. Likewise, other studies suggested that stress might negatively interact with synaptic plasticity (Concerto et al., 2017, 2018). As to depression, a recent study performed by Mineo et al. (2018) showed how cortical excitability might change depending on positive or negative thinking. These studies confirmed that tDCS interventions on stroke patients presenting with stress or depressive symptoms could lead to unpredictable or unusual results. Individual variability is therefore a crucial factor, which can greatly influence the outcomes of a tDCS study.

Generally, it may be concluded that tDCS can be an efficient tool to boost the recovery process after a major brain damage, especially if compared with Speech and Language Therapy, but it cannot be used as the exclusive treatment (Bolognini and Miniussi, 2018).

## Combining Transcranial Stimulation With Rehabilitation Techniques

A successful language recovery can be achieved by pairing specific therapeutic approaches with tDCS. The selection of the appropriate approach depends on the impaired language function (Marangolo et al., 2013b, 2014b; Campana et al., 2015).

In this regard, numerous tDCS studies focused on coupling stimulation techniques with SLT, mostly to improve anomia. Among the many protocols, Kang et al. (2007) carried out a study on patients who received a 5-day standardized speech training coupled with active C-tDCS or sham stimulation. The main outcome measure showed an improvement in the percentage of correct responses and reaction time (RT) on a computerized naming test, for each intervention.

Using the same stimulation protocol (Jung et al., 2011), patients could perform speech therapy according to their aphasia type, specific features, and various stimulation responses. The speech therapy methods used were stimulation with auditory and visual sense, such as *Melodic Intonation Therapy, Visual Action Therapy, and Auditory Comprehension Training*.

Other speech therapy methods were also used, such as context and stimulation word-oriented therapy, therapy for promoting aphasics’ communicative effectiveness to improve communication skills, and practice sessions of speaking using a cognitive therapeutic approach. AQ% (Aphasia Quotient) improvement from pre- to post-therapy was greater in patients with less severe, fluent types of aphasia who received treatment 30 days prior to stroke onset. On this note, speech therapy with tDCS was established as a treatment tool for aphasic patients after stroke. Lower initial severity was associated with better responses (Jung et al., 2011).

Speech Therapy can also be combined with dual or bi-hemispheric stimulation. Lee et al. (2013) administered picture naming and reading tasks during the last 15 min of every tDCS session. This protocol led to a significant improvement in response times, with a significant interaction between time and type of interventions, while no significant changes in verbal fluency were observed after single or dual tDCS. Furthermore, in a similar stimulation protocol (Marangolo et al., 2013a) patients were administered all the standardized language tests at the beginning (baseline; T0) and at the end (T10) of each treatment condition, and 1 week after T10 (follow-up; F/U). The therapy method, coupled with stimulation montage, was similar for all patients. Before the treatment, 126 stimuli (syllables, words and sentences) were auditorily presented, one at a time, through an audiotape for three consecutive days. From T1 to T10 the clinician and the patient were seated face-to-face so that the patient could watch the articulatory movements of the clinician and correctly reproduce it. Patients exhibited a significant recovery not only in terms of better accuracy and speed in articulating the treated stimuli, but also in other language tasks (picture description, noun and verb naming, word repetition, word reading) which persisted in the follow-up session (Marangolo et al., 2013a). De Aguiar et al. (2015a) administered bi-hemispheric stimulation on a group of chronic aphasic patients, combined with ACTION, a training based on *Linguistically Motivated Language Therapy* (Bastiaanse et al., 1997). In this study, treatment was provided at the level of simple, declarative sentences, and a task specifically designed to address movement operations was not included. Since Italian is a morphologically rich language, the Italian adaptation of ACTION includes: lexical level (action naming), syntactic level (sentence completion with infinitive), morphosyntactic level (sentence completion with finite verb in three tenses) and sentence construction with finite verb. Therapy was provided over ten 1-h sessions in each phase, and lasted 2 weeks involving treatment with two different tasks. The structured increasing cues provided to each subject depended on whether the participant produced retrieval errors or morphological errors. 10 healthy volunteers were asked to build sentences that described the picture stimuli. The results showed a significant improvement in verbal production after 10 daily sessions, each lasting 1 h, where the first 20 min were paired with real or sham tDCS. The double-blind study specifically focused on verb inflection and sentence construction.

In a recent study by Stahl et al. (2019), the subjects received intensive SLT consisting of naming CAT and face-to-face communicative-pragmatic therapy, after an extensive baseline screening. These tasks included a variety of situations of everyday life that required verbal and non-verbal skills in social interaction. The treatment was administered in two daily sessions over a period of three consecutive weeks (2 h of daily naming therapy; 30 min of daily communicative-pragmatic therapy; total weekly dosage: 12.5 h). Intensive SLT can relieve symptoms in chronic post-stroke aphasia, but effect sizes are moderate (Breitenstein et al., 2017). The results of the study indicate that intensive SLT combined with A-tDCS on M1 benefits naming and communication abilities in chronic post-stroke aphasia, with medium-to-large effect sizes. Covering both utterance-centered and communicative-pragmatic treatment strategies, the selected SLT methods reflect best-practice guidelines in aphasia rehabilitation.

There is yet no consensus about what could be considered the most efficient language rehabilitation technique for an optimal aphasia recovery (Brady et al., 2016). Guillouët et al. (2020) evaluated the impact of bi-hemispheric tDCS combined with SLT into clinical practice. Specifically, they assessed spontaneous speech improvements in response to open questions in patients with poststroke aphasia. The study did not find any significant improvement. However, the small number of patients, even if similar to most previous studies, and the limited time of therapy, could have impacted the significance of the study (Guillouët et al., 2020).

Several studies have shown tDCS paired with naming tasks specifically designed to improve different linguistic domains, such as verb or noun naming or word retrieval. Amongst the studies administering naming tasks, the majority used pictures, video-clips showing objects handling (Flöel et al., 2011; Fiori et al., 2013; Marangolo et al., 2013b), or written words (Vestito et al., 2014; Marangolo et al., 2017). It was then asked to name each item correctly. For instance, in the study run by Flöel et al. (2011), 12 patients with chronic anomia received 2 h of daily naming CAT across 3 consecutive days. Training involved a decreasing cueing hierarchy with 5 difficulty levels that have been shown to be highly effective to improve naming difficulties. Results demonstrate that short-term high-frequency anomia training has a large effect on naming ability in chronic aphasia that was maintained for at least 2 weeks.

Furthermore, Marangolo et al. (2013b) implemented the use of video-clips. For each subject, the selected items were subdivided into three groups of 34 actions each, matched for frequency and length. Each condition was performed in five consecutive daily sessions over 3 weeks, with 6 days of intersession interval, while the subjects underwent intensive language training to recover their verb naming difficulties. The study suggests that A-tDCS applied over the left frontal region, together with simultaneous intensive language training, led to the greatest amount of verb naming improvement.

The 2013 study conducted by Fiori and colleagues also showed a significant improvement in naming after tDCS paired to naming tasks, using both pictures of objects and video-clips of actions. For each treatment, subjects were asked to name aloud each item that appeared on screen. Generally, the patients were more accurate in the naming of objects. These results allow us to affirm that the recovery in naming objects or actions was related to the stimulation of distinct brain regions. The follow-up testing showed recovery of the two categories at one and four weeks after the end of treatment.

Marangolo et al. (2017) tested the use of written words as a training. The items referred to action verbs and non-manipulable objects. Nouns and actions were matched for number of letters, surface frequency, imageability and age of acquisition. Both imageability and age-of-acquisition ratings were collected by asking volunteers to judge printed words. The outcomes showed a significant improvement more in verbs than noun naming.

Performing word retrieval tasks during tDCS generally seemed to improve picture naming in post-stroke aphasia patients (Fiori et al., 2011). Kang et al. (2011) training consisted of modules, such as answering dichotomic yes/no questions, cued naming about target pictures, and word-picture matching tasks, based on pictures sorted by category. Word-retrieval training was performed while patients were being treated with tDCS or sham stimulation. The main findings of this double blind, sham controlled, crossover study show that this stimulation protocol improved picture naming accuracy while performing word-retrieval training, if compared with sham stimulation. Also, PNT based studies focused on the individual variability resulting from differences in electrode montage (Shah-Basak et al., 2015; Norise et al., 2017). As described in the study from Shah-Basak et al. (2016), during the 20-min active stimulation, subjects completed a picture-naming task that was based on CILT, during which non-verbal communication between subjects and the experimenter was minimized (Pulvermüller et al., 2001; Maher et al., 2006). Subjects with a more severe clinical picture showed greater improvements in verbal fluidity. These improvements were maintained at the 2-week follow-up. Still, picture-word matching tasks were often used in the online tDCS studies (Baker et al., 2010; Rosso et al., 2014; Darkow et al., 2017; Fridriksson et al., 2018a; Pestalozzi et al., 2018).

In a recent study, the word-finding therapy protocol was based on the *Cueing Hierarchy Therapy* (Linebaugh et al., 2005). During picture naming, the therapist used cueing techniques to help the participants retrieve and produce the target words correctly. The cue of low stimulus power was presented first, followed by increasingly powerful cues until the correct word was retrieved and produced. As the relative power of the cues differed across participants with aphasia, the exact cueing hierarchy was personalized (Spielmann et al., 2018).

The implementation of domain-dependent tasks, combined with tDCS, can also rely on repetition tasks. For instance, a clinician can audibly present one stimulus at a time, and for each stimulus, the treatment involves the use of four different steps, which would progressively induce the patient to correctly reproduce the items. Initially, presenting the entire stimulus and asking the patient to repeat it. then presenting the stimuli with a pause between syllables, prolonged vowel sound and exaggerated articulatory gestures, to make the patient repeat the item. The four steps become progressively more difficult. If the patient is not able to articulate the stimulus in the first step, the clinician would move on to the next step and so on up to the last step. The potential of tDCS combined with the intensive repetition training was investigated (Marangolo et al., 2011, 2016). Evidence showed a beneficial effect on the recovery of the aphasic subjects’ articulatory disturbances and shorter RT. Moreover, the follow-up testing revealed retention of the achieved improvement suggesting long-term recovery of the subjects’ articulatory disturbances.

Only one study from our literature focused on investigating motor cortex functional involvement in access to specific lexical-semantic (object vs. action relatedness) information in post-stroke aphasia (Branscheidt et al., 2017). Lexical decision is known to tap into lexical and semantic information (Balota and Chumbley, 1984) and shows very similar brain activation patterns than naming (Carreiras et al., 2007). 130 pseudowords and existing words (verbs related to hand actions and nouns related to objects) were presented to the patients in random sequence on a computer screen. Whereas no specific effects of tDCS on lexical decision latencies were observed, the results showed that anodal stimulation to the MC (Motor Cortex) of the language-dominant hemisphere improved overall accuracy in a lexical decision task. Importantly, improvement in decision accuracy depended on the meaning of the words: lexical decisions were significantly more accurate under anodal stimulation for action words and “action-like” pseudowords (ALP), while object words and “object-like” pseudowords (OLP) were not significantly affected by tDCS (Branscheidt et al., 2017).

Non-invasive stimulation was also combined with CTA. In a study conducted by Marangolo (2013), then replicated in 2014, patients were required to talk about a number of video clips, shown while undergoing left transcranial stimulation. Dialog and sentence production specifically improved more after anodal tDCS on Broca’s area, if compared to Wernicke’s anodal stimulation and the sham control condition. Progresses were maintained at follow-up one month later.

An overlap of linguistic and vocal networks is shown both in the right and the left hemisphere (Özdemir et al., 2006; Racette et al., 2006). Singing and intonation can be meaningful resources in case of a left-brain damage, and can help facilitate language recovery by employing the healthy right hemisphere (Vines et al., 2009). Melodic intonation therapy, by using prosody and melody, might therefore be a valid alternative to classic tasks paired with tDCS. It emphasizes the rhythmic and melodic elements of language and it is based on clinical observations that showed that several aphasic patients were often able to sing song lyrics better than they could pronounce them. MIT uses simplified prosody and the words are intoned slowly syllable by syllable. Its validity might be related to speech-specific brain regions in both hemispheres, but it is still not clear which areas drive the therapeutic effect of MIT (Pani et al., 2016). It is thought that the right posterior frontal gyrus may play a role in the recovery process linked to melodic training (Vines et al., 2009). Vines et al. (2011) in fact showed a significant improvement in verbal fluency after right anodal stimulation paired with MIT. Cipollari et al. (2015) showed similar results in their study.

However, given the wide clinical and neurological variability among aphasic patients, it is unlikely that a single therapeutic procedure can be universally effective. Nevertheless, interventions’ variety shows how many cortical and functional networks can be exploited for linguistic rehabilitation.

## Action Observation in Aphasia Rehabilitation

Gestures and action observation can be a powerful tool in rehabilitation. Studies showed a meaningful relationship between gestures and the processing of communicative intention (Krönke et al., 2013). Combining gestures with verbal production as a rehabilitation technique can have therapeutic effects on aphasic patients (Hanlon et al., 1990). For instance, Richards et al. (2002) argued that by using their non-dominant hand to mimic a gesture, patients would enhance their linguistic skills. Hence, action observation might be useful in improving naming, especially verbs (Rodriguez et al., 2006). The hypothesis underlying AOT is that the observation and imitation of a gesture made by a third person may promote a faster and more effective recovery of motor difficulties (Ertelt et al., 2007) and of language deficits (Small and Llano, 2009; Rose, 2013; Marangolo and Caltagirone, 2014). In this way, language is strictly interconnected with gestures, based on the “motor theory of speech perception” by Liberman and colleagues (1967); Liberman and Mattingly (1985), which considered language in terms of how it is produced, rather than how it sounds, so that perceiving speech corresponds to perceiving vocal tract gestures.

Among the AOT studies, Marangolo and colleagues identified intensive language treatment, based on action observation and execution, as a leading approach to a significant increase in verb production (Marangolo et al., 2010, 2012) and spontaneous speech (Marangolo et al., 2014a). It is therefore possible to suggest that the simple systematic and repeated observation of actions can be an effective and alternative therapeutic strategy for the recovery of words in aphasic subjects (Marangolo et al., 2012).

About the action imitation, Lee et al. (2010) developed a computerized treatment for aphasia called IMITATE (*Intensive Mouth Imitation and Talking for Aphasia Therapeutic Effect*), based on the observation of “the act of speaking.” The therapeutic protocol consists of a first phase characterized by the observation of short video-clips of actors pronouncing words, verbs or phrases aloud. This is followed by a second phase in which subjects are asked to repeat what they have seen and heard in the previous phase. The high-intensity training requires 90 min a day for six weeks. IMITATE generates a gradual learning, enabled by a progressive complexity of the presented stimuli. In fact, the level of difficulty increases weekly, starting from monosyllabic words, and then moving on to bisyllabic, trisyllabic and finally to sentences. The effects of this treatment were investigated in a RCT, and first results showed a significant increase in production capacity in the experimental group. The study conducted by Duncan and Small (2017) used IMITATE to investigate the effects of an intensive treatment based on imitation on narrative ability. After six weeks of intensive treatment, the results showed an important increase of both measures considered and a substantial improvement in narrative ability.

In 2018, Zettin and colleagues helped expand the AOT literature by administering the IMITAF rehabilitation protocol, referring to the work of Lee et al. (2010). Seven subjects with chronic aphasia resulting from a brain injury were enrolled in the study. The within-subject design protocol lasted three months and included the administration of two treatments. Firstly, a month of traditional SLT was planned (T0), then the IMITAF training started. Each rehabilitation phase lasted 30 days and was divided into three daily sessions per week. During every IMITAF training session, each participant sat in front of a computer screen, and was asked to observe six actors while pronouncing different words or short sentences. After the observation of the actors, participants were asked to repeat what they heard. Unlike IMITATE (Lee et al., 2010), in which levels of difficulty are progressive, during IMITAF the shift from one level to another of the program is decided according to the subjects’ needs, their response to the treatment and their speed of improvement. More specifically, the transition to the next level could occur only when the patient showed familiarity with the prior level. AOT protocol requires playing an action video-clip on a computer screen (Bonifazi et al., 2013). The patient can perform computer-based treatments without the help of a therapist or caregiver (Zettin et al., 2018). For these reasons, the treatment could be performed at the rehabilitation center or at home, depending on the patient’s needs. The results of the pilot study by Zettin et al. (2018) showed a significant post-treatment decrease in naming difficulties for the entire sample. Participants showed improvements in retrieving words without phonemic or semantic cues, a better repetition and naming ability.

As emerged from the outcomes of the study here mentioned, the global coherence analysis represents a suitable measure to be used in clinical practice. Taken together, treatment on action imitation may contribute to the reduction of anomia following aphasia, in accordance with previous research (Lee et al., 2010; Marangolo et al., 2010, 2012; Bonifazi et al., 2013).

## Discussion

The main aim of this review is to give an overview of the state of the art of tDCS treatments for aphasia. This brain stimulation technique has been proven to be effective in promoting a successful recovery in both the short and long term after a brain injury. When used in combination with treatments such as SLT, CIAT or Intensive Action Treatment, tDCS has generally promoted a better recovery of the impaired functions, if compared with offline tDCS (De Aguiar et al., 2015b; Berube and Hillis, 2019). Furthermore, tDCS paired with naming tasks specifically designed to improve different linguistic domains (i.e., verb/noun naming or word retrieval) have shown significant results. The majority of the studies requiring domain-dependent tasks used pictures, video-clips showing objects handling, or written words (Flöel et al., 2011; Fiori et al., 2013; Marangolo et al., 2013a,b). Both the task-induced synaptic activity and the cortical activity generated by tDCS simultaneously contributed to a better recovery (De Aguiar et al., 2015b). However, the tasks paired with tDCS should not be too difficult, as the excessive neural noise could interfere with the rehabilitative process and the training could be less beneficial (Miniussi et al., 2013). As an alternative to classic tasks combined with tDCS, MIT appears to be a promising tool to facilitate verbal fluency recovery by using melody and prosody (Sparks, 2008; Vines et al., 2009; Cipollari et al., 2015; Pani et al., 2016).

In addition to the traditional rehabilitation protocols, Action Observation Therapy, such as IMITATE and its Italian adaptation IMITAF, may contribute to the reduction of anomia following post-stroke aphasia in clinical practice, by activating both frontal and parietal brain regions and the mirror neurons systems (Chapey, 2008; Lee et al., 2010; Duncan and Small, 2017; Zettin et al., 2018). The potential of combining such techniques with tDCS would therefore be a possibility of further improvement, also providing the clinician with a new intervention tool. The association of a tDCS protocol with a dedicated rehabilitation training would favor a generalized long-term improvement of the different components of language.

As for the electrode montage, the evidence would suggest a better efficacy of perilesional anodal stimulation, especially in cases of moderate damage and at a chronic stage (Schlaug et al., 2011; De Aguiar et al., 2015b; Bucur and Papagno, 2019). Rather, for more severe injuries, anodal stimulation of the homolog areas of the healthy hemisphere is recommended (Schlaug et al., 2011; AlHarbi et al., 2017). Right cathodal tDCS might be useful to reduce the interference of the healthy hemisphere, when residual perilesional activity is shown (Kang et al., 2011). In contrast, it can be dysfunctional when the healthy hemisphere partially takes over the impaired language functions to counterbalance the damage (Rosso et al., 2014).

For both safety and methodological issues, as reported in all tDCS studies examined, it is advisable to administer tDCS for no longer than 30 min per session, at 1 or 2 mA (De Aguiar et al., 2015b). It is also recommended to opt for 5 to 15 sessions of active stimulation. As for the aphasia treatments paired with tDCS, it is advisable to perform them concurrently with the stimulation. Since studies using modality-dependent tasks instead of behavioral treatments showed the best results, the implementation of these specific tasks should be encouraged (Vines et al., 2011; Marangolo et al., 2013b; Biou et al., 2019). However, maximal language gains could be obtained by differentiating task, length, and number of sessions according to patients’ condition and needs. Several studies included in this review also comprehended a follow-up examination of the patients’ progress, essential to detect the long-term effects of tDCS (Fiori et al., 2011, 2013, 2019; Fridriksson et al., 2011; Marangolo et al., 2013a,b; Cipollari et al., 2015; Shah-Basak et al., 2015; Basat et al., 2016; Woodhead et al., 2018).

However, future studies should consider other crucial elements, such as the necessity to include large randomized controlled trials to monitor patients’ progresses over time. The reduced sample size found in the majority of tDCS studies has made it more difficult to draw more in-depth conclusions about the efficacy of a stimulation parameter. In addition, as reported by several authors (Miniussi et al., 2013; Rosso et al., 2014), while using standardized tests for the assessment of patients’ language abilities, it would be better to compare multiple samples at the same time, thus reducing the heterogeneity of the outcomes. This would also make it easier to replicate a study protocol. Several studies identified valid tDCS protocols that, if implemented on other samples, would likewise lead to significant improvements in both the short and long term (Meinzer et al., 2016; Fridriksson et al., 2018b; Woodhead et al., 2018; Stahl et al., 2019). A major therapeutic goal is to prove that patients retain the acquired skills over time, and that the treatment can work in real-world settings too. Thus, standardized tests assessing daily communication skills could be good indicators of a functional improvement after a tDCS study (Miniussi et al., 2013). Using the same tests on different samples would guarantee a clearer understanding of different results.

Another aspect that requires further investigation is the identification of the most suitable patients for this type of intervention. Currently, the small size does not allow to clarify this issue. Moreover, all tDCS studies here reported have not compared groups of patients with different deficits (i.e., patients with both comprehension and production deficits vs patients with only production deficits) and/or with different severities of the same linguistic domains (i.e., patients with severe comprehension deficits vs less severe patients). It should in fact be considered that aphasia can occur in several different ways and can lead to different impairment degrees. Additionally, patients with the same initial deficit could still show different recovery times and reactions to the same therapy (Ulanov et al., 2019). Thus, finding a large homogeneous sample and drawing universal conclusions from the outcome of a study can be complex and often unfeasible.

As for the configuration of the brain injury, the right parameters and stimulation site should ideally be selected according to the patient’s individual characteristics (Vines et al., 2009, 2011; Rosso et al., 2014). The implementation of neuroimaging techniques, such as fMRI, help the clinicians to better tailor the treatment. For example, functional neuroimaging would help locate the exact area of the lesion for a better positioning of the electrodes (Marshall et al., 2000; Baker et al., 2010). Additionally, the areas of neural activity spared by the damage during the execution of specific tasks would be better identified. A tailored stimulation would therefore optimize the patient’s recovery. However, it must be stated that, although a better outcome is more likely, using functional imaging would slow down the treatment process, consequently increasing the cost of therapy. Therefore, this may not always be appropriate or feasible in clinical practice where timing and accessibility are essential. Since literature on this issue is lacking, future experimental designs should contemplate personalized rehabilitation protocols combining tDCS with IMITAF (Zettin et al., 2018). Furthermore, the rehabilitation potential of combining tDCS with IMITAF on patients with chronic aphasia due to an acquired brain injury should be investigated in order to ascertain whether long-term reduction in aphasic symptoms and an improvement in naming would occur.

Based on the results of the studies here presented, a new clinical trial for acquired chronic aphasia rehabilitation can be suggested. According to our ongoing project, the enrolled patients will be divided into two groups: experimental and control. The first group will undergo perilesional anodal stimulation, while the second one will receive a sham stimulation. The active anodal stimulation will be administered at 1.5 mA for the first 20 min of each IMITAF rehabilitation session. The IMITAF training will have a total duration of 6 weeks (5 days per week, weekends off). The underlying idea is that the constant and repetitive application of tDCS might favor a significant decrease in aphasic symptoms if compared to traditional techniques. Short-and long-term improvements are also expected in the patients treated.

## Author Contributions

MZ, MV, CB, and GN: article research and selection and text conception and editing. MV: primary writing of aphasia treatments and rehabilitation techniques. CB: primary writing of patient selection and discussion. GN: primary writing of tDCS techniques. DD: primary writing of introduction and editing. All authors contributed to the article and approved the submitted version.

## Conflict of Interest

The authors declare that the research was conducted in the absence of any commercial or financial relationships that could be construed as a potential conflict of interest.

## Publisher’s Note

All claims expressed in this article are solely those of the authors and do not necessarily represent those of their affiliated organizations, or those of the publisher, the editors and the reviewers. Any product that may be evaluated in this article, or claim that may be made by its manufacturer, is not guaranteed or endorsed by the publisher.

## References

[B1] Abraham W. C. Philpot B. (2009). Metaplasticity. *Scholarpedia* 4:4894.

[B2] Albert M. L. Sparks R. W. Helm N. A. (1973). Melodic intonation therapy for aphasia. *Arch. Neurol.* 29 130–131. 10.1001/archneur.1973.00490260074018 4717723

[B3] AlHarbi M. F. Armijo-Olivo S. Kim E. S. (2017). Transcranial direct current stimulation (TDCS) to improve naming ability in post-stroke aphasia: a critical review. *Behav. Brain Res.* 332 7–15. 10.1016/j.bbr.2017.05.050 28572057

[B4] Ardolino G. Bossi B. Barbieri S. Priori A. (2005). Non-synaptic mechanisms underlie the after-effects of cathodal transcutaneous direct current stimulation of the human brain. *J. Physiol.* 568 653–663. 10.1113/jphysiol.2005.088310 16037080 PMC1474743

[B5] Baker J. M. Rorden C. Fridriksson J. (2010). Using transcranial direct-current stimulation to treat stroke patients with aphasia. *Stroke* 41 1229–1236. 10.1161/STROKEAHA.109.576785 20395612 PMC2876210

[B6] Balota D. A. Chumbley J. I. (1984). Are lexical decisions a good measure of lexical access? The role of word frequency in the neglected decision stage. *J. Exp. Psychol. Hum. Percept. Perform.* 10:340. 10.1037//0096-1523.10.3.340 6242411

[B7] Barbati S. A. Cocco S. Longo V. Spinelli M. Gironi K. Mattera A. (2020). Enhancing plasticity mechanisms in the mouse motor cortex by anodal transcranial direct-current stimulation: the contribution of nitric oxide signaling. *Cereb. Cortex* 30 2972–2985. 10.1093/cercor/bhz288 31821409

[B8] Barlow T. (1877). On the case of double cerebral hemiplegia, with cerebral symmetrical lesions. *Br. Med. J.* 2 103–104. 10.1136/bmj.2.865.103 20748595 PMC2221124

[B9] Basat A. L. B. Gvion A. Vatine J. J. Mashal N. (2016). Transcranial direct current stimulation to improve naming abilities of persons with chronic aphasia: a preliminary study using individualized based protocol. *J. Neurolinguist.* 38 1–13. 10.1016/j.jneuroling.2015.09.004

[B10] Basso A. (2003). *Aphasia and Its Therapy.* Oxford: Oxford University Press.

[B11] Basso A. Gardelli M. Grasi M. P. Mariotti M. (1989). The role of the right hemisphere in recovery from aphasia: two case studies. *Cortex* 25 555–566. 10.1016/s0010-9452(89)80017-6 2612175

[B12] Bastiaanse R. Jonkers R. Quak C. H. Varela Put M. (1997). *Werkwoordproductie op Woorden Zinsniveau [Verb Production at the Word and Sentence Level].* Lisse: Swets Test Publishers.

[B13] Batsikadze G. Moliadze V. Paulus W. Kuo M. F. Nitsche M. A. (2013). Partially non-linear stimulation intensity-dependent effects of direct current stimulation on motor cortex excitability in humans. *J. Physiol.* 591 1987–2000. 10.1113/jphysiol.2012.249730 23339180 PMC3624864

[B14] Berube S. Hillis A. E. (2019). Advances and innovations in aphasia treatment trials. *Stroke* 50 2977–2984. 10.1161/STROKEAHA.119.025290 31510904 PMC6756955

[B15] Beukelman D. R. (1985). *Augmentative Communication Strategies for Adults with Acute or Chronic Medical Conditions.* Baltimore, MD: Paul H. Brookes Publishing Co.

[B16] Biou E. Cassoudesalle H. Cogné M. Sibon I. De Gabory I. Dehail P. (2019). Transcranial direct current stimulation in post-stroke aphasia rehabilitation: a systematic review. *Ann. Phys. Rehabil. Med.* 62 104–121. 10.1016/j.rehab.2019.01.003 30660671

[B17] Bolognini N. Miniussi C. (2018). Noninvasive brain stimulation of the parietal lobe for improving neurologic, neuropsychologic, and neuropsychiatric deficits. *Handb. Clin. Neurol.* 151 427–446. 10.1016/B978-0-444-63622-5.00022-X 29519473

[B18] Bonifazi S. Tomaiuolo F. Altoe’ G. Ceravolo M. G. Provinciali L. Marangolo P. (2013). Action Observation as a useful approach for enhancing recovery of verb production: new evidence from aphasia. *Eur. J. Phys. Rehabil. Med.* 49 1–9. 23486304

[B19] Brady M. C. Kelly H. Godwin J. Enderby P. Campbell P. (2016). Speech and language therapy for aphasia following stroke. *Cochrane Database Syst. Rev.* 2016:CD000425. 10.1002/14651858.CD000425.pub4 27245310 PMC8078645

[B20] Branscheidt M. Hoppe J. Zwitserlood P. Liuzzi G. (2017). TDCS over the motor cortex improves lexical retrieval of action words in poststroke aphasia. *J. Neurophysiol.* 119 621–630. 10.1152/jn.00285.2017 29070627

[B21] Breitenstein C. Grewe T. Flöel A. Ziegler W. Springer L. Martus P. (2017). Intensive speech and language therapy in patients with chronic aphasia after stroke: a randomised, open-label, blinded-endpoint, controlled trial in a health-care setting. *The Lancet* 389 1528–1538. 10.1016/S0140-6736(17)30067-3 28256356

[B22] Brennan D. M. Georgeadis A. C. Baron C. R. Barker L. M. (2004). The effect of videoconference-based telerehabilitation on story retelling performance by brain injured subjects and its implications for remote speech-language therapy. *Telemed. J. EHealth* 10 147–154. 10.1089/tmj.2004.10.147 15319044

[B23] Buckner R. L. Corbetta M. Schatz J. Raichle M. E. Petersen S. E. (1996). Preserved speech abilities and compensation following prefrontal damage. *Proc. Natl. Acad. Sci. U.S.A.* 93 1249–1253. 10.1073/pnas.93.3.1249 8577749 PMC40065

[B24] Bucur M. Papagno C. (2019). Are transcranial brain stimulation effects long-lasting in post-stroke aphasia? A comparative systematic review and meta-analysis on naming performance. *Neurosci. Biobehav. Rev.* 102 264–289. 10.1016/j.neubiorev.2019.04.019 31077693

[B25] Bütefisch C. M. Weβling M. Netz J. Seitz R. J. Hömberg V. (2008). Relationship between interhemispheric inhibition and motor cortex excitability in subacute stroke patients. *Neurorehabil. Neural Repair* 22 4–21. 10.1177/1545968307301769 17507644

[B26] Byng S. Coltheart M. (1986). Aphasia therapy research: methodological requirements and illustrative results. *Adv. Psychol.* 34 191–213.

[B27] Cambiaghi M. Cherchi L. Masin L. Infortuna C. Briski N. Caviasco C. (2021). High-frequency repetitive transcranial magnetic stimulation enhances layer II/III morphological dendritic plasticity in mouse primary motor cortex. *Behav. Brain Res.* 410:113352. 10.1016/j.bbr.2021.113352 33979657

[B28] Cambiaghi M. Crupi R. Bautista E. L. Elsamadisi A. Malik W. Pozdniakova H. (2020). The effects of 1-Hz rTMS on emotional behavior and dendritic complexity of mature and newly generated dentate gyrus neurons in male mice. *Int. J. Environ. Res. Public Health* 17:4074. 10.3390/ijerph17114074 32521613 PMC7312937

[B29] Campana S. Caltagirone C. Marangolo P. (2015). Combining voxel-based lesion-symptom mapping (VLSM) with A-tDCS language treatment: predicting outcome of recovery in nonfluent chronic aphasia. *Brain Stimul.* 8 769–776. 10.1016/j.brs.2015.01.413 25732786

[B30] Cantone M. Lanza G. Ranieri F. Opie G. M. Terranova C. (2021). Non-invasive brain stimulation in the study and modulation of metaplasticity in neurological disorders. *Front. Neurol.* 12:721906. 10.3389/fneur.2021.721906 34276553 PMC8277922

[B31] Carreiras M. Mechelli A. Estévez A. Price C. J. (2007). Brain activation for lexical decision and reading aloud: two sides of the same coin?. *J. Cogn. Neurosci.* 19 433–444. 10.1162/jocn.2007.19.3.433 17335392

[B32] Cassidy J. M. Cramer S. C. (2017). Spontaneous and therapeutic-induced mechanisms of functional recovery after stroke. *Transl. Stroke Res.* 8 33–46. 10.1007/s12975-016-0467-5 27109642 PMC5079852

[B33] Caumo W. Souza I. C. Torres I. L. Medeiros L. Souza A. Deitos A. (2012). Neurobiological effects of transcranial direct current stimulation: a review. *Front. Psychiatry* 3:110. 10.3389/fpsyt.2012.00110 23293607 PMC3531595

[B34] Chapey R. (2008). *Language Intervention Strategies in Aphasia and Related Neurogenic Communication Disorders*, 5th Edn. Baltimore, MD: Lippincott Williams & Wilkins.

[B35] Chase H. W. Boudewyn M. A. Carter C. S. Phillips M. L. (2020). Transcranial direct current stimulation: a roadmap for research, from mechanism of action to clinical implementation. *Mol. Psychiatry* 25 397–407. 10.1038/s41380-019-0499-9 31455860 PMC6981019

[B36] Cheng W. Li Y. Cheng B. Chen Y. Chen Z. Cui L. (2021). Effects of transcranial direct current stimulation over the right hemisphere on naming ability in patients with poststroke aphasia: a meta-analysis. *J. Neurolinguist.* 58:100986. 10.1016/j.jneuroling.2021.100986

[B37] Cherney L. R. Babbitt E. M. Wang X. Pitts L. L. (2021). Extended fMRI-guided anodal and cathodal transcranial direct current stimulation targeting perilesional areas in post-stroke aphasia: a pilot randomized clinical trial. *Brain Sci.* 11:306. 10.3390/brainsci11030306 33671031 PMC7997197

[B38] Cipollari S. Veniero D. Razzano C. Caltagirone C. Koch G. Marangolo P. (2015). Combining TMS-EEG with transcranial direct current stimulation language treatment in aphasia. *Expert Rev. Neurother.* 15 833–845. 10.1586/14737175.2015.1049998 26109229

[B39] Citri A. Malenka R. C. (2008). Synaptic plasticity: multiple forms, functions, and mechanisms. *Neuropsychopharmacology* 33 18–41. 10.1038/sj.npp.1301559 17728696

[B40] Concerto C. Infortuna C. Muscatello M. R. A. Bruno A. Zoccali R. Chusid E. (2018). Exploring the effect of adaptogenic Rhodiola Rosea extract on neuroplasticity in humans. *Complement. Ther. Med.* 41 141–146. 10.1016/j.ctim.2018.09.013 30477830

[B41] Concerto C. Patel D. Infortuna C. Chusid E. Muscatello M. R. Bruno A. (2017). Academic stress disrupts cortical plasticity in graduate students. *Stress* 20 212–216. 10.1080/10253890.2017.1301424 28320257

[B42] Cramer S. C. (2018). Stimulating dialogue through treatment of poststroke aphasia with transcranial direct current stimulation. *JAMA Neurol.* 75 1465–1467. 10.1001/jamaneurol.2018.1751 30128556

[B43] Crinion J. T. (2016). Transcranial direct current stimulation as a novel method for enhancing aphasia treatment effects. *Eur. Psychol.* 21 65–77. 10.1027/1016-9040/a000254

[B44] Crosson B. McGregor K. Gopinath K. S. Conway T. W. Benjamin M. Chang Y. L. (2007). Functional MRI of language in aphasia: a review of the literature and the methodological challenges. *Neuropsychol. Rev.* 17 157–177. 10.1007/s11065-007-9024-z 17525865 PMC2659355

[B45] Darkow R. Martin A. Würtz A. Flöel A. Meinzer M. (2017). Transcranial direct current stimulation effects on neural processing in post-stroke aphasia. *Hum. Brain Mapp.* 38 1518–1531. 10.1002/hbm.23469 27859982 PMC6866985

[B46] De Aguiar V. Bastiaanse R. Capasso R. Gandolfi M. Smania N. Rossi G. (2015a). Can tDCS enhance item-specific effects and generalization after linguistically motivated aphasia therapy for verbs? *Front. Behav. Neurosci.* 9:190. 10.3389/fnbeh.2015.00190 26903832 PMC4519773

[B47] De Aguiar V. Paolazzi C. L. Miceli G. (2015b). tDCS in post-stroke aphasia: the role of stimulation parameters, behavioral treatment and patient characteristics. *Cortex* 63 296–316. 10.1016/j.cortex.2014.08.015 25460496

[B48] De Luca R. Aragona B. Leonardi S. Torrisi M. Galletti B. Galletti F. (2018). Computerized training in poststroke aphasia: what about the long-term effects? A randomized clinical trial. *J. Stroke Cerebrovasc. Dis.* 27 2271–2276. 10.1016/j.jstrokecerebrovasdis.2018.04.019 29880209

[B49] Doogan C. Dignam J. Copland D. Leff A. (2018). Aphasia recovery: when how and who to treat? *Curr. Neurol. Neurosci. Rep.* 18 1–7. 10.1007/s11910-018-0891-x 30324233 PMC6209017

[B50] Duncan E. S. Small S. L. (2017). Imitation-based aphasia therapy increases narrative content: a case series. *Clin. Rehabil.* 31 1500–1507. 10.1177/0269215517703765 28393551 PMC8668037

[B51] Ertelt D. Small S. Solodkin A. Dettmers C. McNamara A. Binkofski F. (2007). Action observation has a positive impact on rehabilitation of motor deficits after stroke. *NeuroImage* 36 164–173. 10.1016/j.neuroimage.2007.03.043 17499164

[B52] Fiori V. Cipollari S. Di Paola M. Razzano C. Caltagirone C. Marangolo P. (2013). TDCS stimulation segregates words in the brain: evidence from aphasia. *Front. Hum. Neurosci.* 7:269. 10.3389/fnhum.2013.00269 23785323 PMC3682157

[B53] Fiori V. Coccia M. Marinelli C. V. Vecchi V. Bonifazi S. Ceravolo M. G. (2011). Transcranial direct current stimulation improves word retrieval in healthy and nonfluent aphasic subjects. *J. Cogn. Neurosci.* 23 2309–2323. 10.1162/jocn.2010.21579 20946060

[B54] Fiori V. Nitsche M. A. Cucuzza G. Caltagirone C. Marangolo P. (2019). High-definition transcranial direct current stimulation improves verb recovery in aphasic patients depending on current intensity. *Neuroscience* 406 159–166. 10.1016/j.neuroscience.2019.03.010 30876982

[B55] Fisicaro F. Lanza G. Bella R. Pennisi M. (2020). “Self-neuroenhancement”: the last frontier of noninvasive brain stimulation? *J. Clin. Neurol.* 16 158–159. 31942774 10.3988/jcn.2020.16.1.158 PMC6974812

[B56] Fisicaro F. Lanza G. Grasso A. A. Pennisi G. Bella R. Paulus W. (2019). Repetitive transcranial magnetic stimulation in stroke rehabilitation: review of the current evidence and pitfalls. *Ther. Adv. Neurol. Disord.* 12:1756286419878317. 10.1177/1756286419878317 PMC6763938 31598137

[B57] Flöel A. Meinzer M. Kirstein R. Nijhof S. Deppe M. Knecht S. (2011). Short-term anomia training and electrical brain stimulation. *Stroke* 42 2065–2067. 10.1161/STROKEAHA.110.609032 21636820

[B58] Fricke K. Seeber A. A. Thirugnanasambandam N. Paulus W. Nitsche M. A. Rothwell J. C. (2011). Time course of the induction of homeostatic plasticity generated by repeated transcranial direct current stimulation of the human motor cortex. *J. Neurophysiol.* 105 1141–1149. 10.3233/RNN-170783 21177994

[B59] Fridriksson J. (2011). Measuring and inducing brain plasticity in chronic aphasia. *J. Commun. Disord.* 44 557–563. 10.1016/j.jcomdis.2011.04.009 21620414 PMC3162133

[B60] Fridriksson J. Hubbard H. I. Hudspeth S. G. (2012). Transcranial brain stimulation to treat aphasia: a clinical perspective. *Semin. Speech Lang.* 33 188–202. 10.1055/s-0032-1320039 22851341

[B61] Fridriksson J. Richardson J. D. Baker J. M. Rorden C. (2011). Transcranial direct current stimulation improves naming reaction time in fluent aphasia: a double-blind, sham-controlled study. *Stroke* 42 819–821. 10.1161/STROKEAHA.110.600288 21233468 PMC8210639

[B62] Fridriksson J. Rorden C. Elm J. Sen S. George M. S. Bonilha L. (2018b). Transcranial direct current stimulation vs sham stimulation to treat aphasia after stroke: a randomized clinical trial. *JAMA Neurol.* 75 1470–1476. 10.1001/jamaneurol.2018.2287 30128538 PMC6583191

[B63] Fridriksson J. Elm J. Stark B. C. Basilakos A. Rorden C. Sen S. (2018a). BDNF genotype and TDCS interaction in aphasia treatment. *Brain Stimul.* 11 1276–1281. 10.1016/j.brs.2018.08.009 30150003 PMC6293970

[B64] Galletta E. E. Conner P. Vogel-Eyny A. Marangolo P. (2016). Use of tDCS in aphasia rehabilitation: a systematic review of the behavioral interventions implemented with noninvasive brain stimulation for language recovery. *Am. J. Speech Lang. Pathol.* 25 S854–S867. 10.1044/2016_AJSLP-15-0133 27997958

[B65] Garrett K. Beukelman D. R. (1998). “Adults with severe aphasia,” in *Augmentative and Alternative Communication: Management of Severe Communication Disorders in Children and Adults*, 2nd Edn, eds Beukelman D. R. Mirenda P. (Baltimore, MD: Paul H. Brookes), 465–499.

[B66] Gold B. T. Kertesz A. (2000). Right hemisphere semantic processing of visual words in an aphasic patient: an fMRI study. *Brain Lang.* 73 456–465. 10.1006/brln.2000.2317 10860566

[B67] Guillouët E. Cogné M. Saverot E. Roche N. Pradat-Diehl P. Weill-Chounlamountry A. (2020). Impact of combined transcranial direct current stimulation and speech-language therapy on spontaneous speech in aphasia: a randomized controlled double-blind study. *J. Int. Neuropsychol. Soc.* 26 7–18. 10.1017/S1355617719001036 31983371

[B68] Hamilton R. H. Chrysikou E. G. Coslett B. (2011). Mechanisms of aphasia recovery after stroke and the role of noninvasive brain stimulation. *Brain Lang.* 118 40–50. 10.1016/j.bandl.2011.02.005 21459427 PMC3109088

[B69] Hanlon R. E. Brown J. W. Gerstman L. J. (1990). Enhancement of naming in nonfluent aphasia through gesture. *Brain Lang.* 38 298–314. 10.1016/0093-934X(90)90116-X 2322814

[B70] Hebb D. O. (2005). *The Organization of Behavior: A Neuropsychological Theory.* Hove: Psychology Press.

[B71] Heiss W. D. Thiel A. (2006). A proposed regional hierarchy in recovery of post-stroke aphasia. *Brain Lang.* 98 118–123. 10.1016/j.bandl.2006.02.002 16564566

[B72] Helm-Estabrooks N. Albert M. L. (1991). *Manual of Aphasia Therapy.* Austin, TX: Pro-Ed.

[B73] Hillis A. E. (1998). Treatment of naming disorders: new issues regarding old therapies. *J. Int. Neuropsychol. Soc.* 4 648–660. 10.1017/S135561779846613X 10050369

[B74] Holland R. Crinion J. (2012). Can tDCS enhance treatment of aphasia after stroke? *Aphasiology* 26 1169–1191. 10.1080/02687038.2011.616925 23060684 PMC3464450

[B75] Ihara A. S. Miyazaki A. Izawa Y. Takayama M. Hanayama K. Tanemura J. (2020). Enhancement of facilitation training for aphasia by transcranial direct current stimulation. *Front. Hum. Neurosci.* 14:573459. 10.3389/fnhum.2020.573459 33024429 PMC7516201

[B76] Infortuna C. Mineo L. Buffer S. Thomas F. P. Muscatello M. R. A. Aguglia E. (2021). Acute social and somatic stress alters cortical metaplasticity probed with non-invasive brain stimulation in humans. *Int. J. Psychophysiol.* 170 1–5. 10.1016/j.ijpsycho.2021.09.004 34547303

[B77] Jacquemot C. Dupoux E. Robotham L. Bachoud-Lévi A.-C. (2012). Specificity in rehabilitation of word production: a meta-analysis and a case study. *Behav. Neurol.* 25 73–101. 10.3233/BEN-2012-0358 22425722 PMC5294258

[B78] Jung I. Y. Lim J. Y. Kang E. K. Sohn H. M. Paik N. J. (2011). The factors associated with good responses to speech therapy combined with transcranial direct current stimulation in post-stroke aphasic patients. *Ann. Rehabil. Med.* 35:460. 10.5535/arm.2011.35.4.460 22506160 PMC3309227

[B79] Kang E. K. Kim Y. K. Sohn H. M. Cohen L. G. Paik N. J. (2011). Improved picture naming in aphasia patients treated with cathodal tDCS to inhibit the right Broca’s homologue area. *Restor. Neurol. Neurosci.* 29 141–152. 10.3233/RNN-2011-0587 21586821 PMC4886370

[B80] Kang E. K. Sohn H. M. Oh M. K. Oh B. M. Jeon J. Y. Kim D. Y. (2007). Paradoxical facilitatory effect of cathodal transcranial direct current stimulation on poststroke aphasia. *Arch. Phys. Med. Rehabil.* 88:e8. 10.1016/j.apmr.2007.08.044

[B81] Khazen T. Shrivastava K. Jada R. Hatoum O. A. Maroun M. (2018). Different mechanisms underlie stress-induced changes in plasticity and metaplasticity in the prefrontal cortex of juvenile and adult animals: emotional-induced metaplasticity in the prefrontal cortex. *Neurobiol. Learn. Mem.* 154 5–11. 10.1016/j.nlm.2018.02.011 29438741

[B82] Kim J. J. Diamond D. M. (2002). The stressed hippocampus, synaptic plasticity and lost memories. *Nat. Rev. Neurosci.* 3 453–462. 10.1038/nrn849 12042880

[B83] Krönke K. M. Mueller K. Friederici A. D. Obrig H. (2013). Learning by doing? The effect of gestures on implicit retrieval of newly acquired words. *Cortex* 49 2553–2568. 10.1016/j.cortex.2012.11.016 23357203

[B84] Laver K. E. Lange B. George S. Deutsch J. E. Saposnik G. Crotty M. (2017). Virtual reality for stroke rehabilitation. *Cochrane Database Syst. Rev.* 11:CD008349. 10.1002/14651858.CD008349.pub4 29156493 PMC6485957

[B85] Lee J. Fowler R. Rodney D. Cherney L. Small S. L. (2010). IMITATE: an intensive computer-based treatment for aphasia based on action observation and imitation. *Aphasiology* 24 449–465. 20543997 10.1080/02687030802714157 PMC2882655

[B86] Lee S. Y. Cheon H. J. Yoon K. J. Chang W. H. Kim Y. H. (2013). Effects of dual transcranial direct current stimulation for aphasia in chronic stroke patients. *Ann. Rehabil. Med.* 37:603. 10.5535/arm.2013.37.5.603 24233579 PMC3825935

[B87] Lefaucheur J. P. Antal A. Ayache S. S. Benninger D. H. Brunelin J. Cogiamanian F. (2017). Evidence-based guidelines on the therapeutic use of transcranial direct current stimulation (tDCS). *Clin. Neurophysiol.* 128 56–92. 10.1016/j.clinph.2016.10.087 27866120

[B88] Liberman A. M. Cooper F. S. Shankweiler D. P. Studdert-Kennedy M. (1967). Perception of the speech code. *Psychol. Rev.* 74:431. 10.1037/h0020279 4170865

[B89] Liberman A. M. Mattingly I. G. (1985). The motor theory of speech perception revised. *Cognition* 21 1–36. 10.1016/0010-0277(85)90021-6 4075760

[B90] Liebetanz D. Nitsche M. A. Tergau F. Paulus W. (2002). Pharmacological approach to the mechanisms of transcranial DC-stimulation-induced after-effects of human motor cortex excitability. *Brain* 125 2238–2247. 10.1093/brain/awf238 12244081

[B91] Liepert J. Storch P. Fritsch A. Weiller C. (2000). Motor cortex disinhibition in acute stroke. *Clin. Neurophysiol.* 111 671–676. 10.1016/S1388-2457(99)00312-0 10727918

[B92] Linebaugh C. W. Shisler R. J. Lehner L. (2005). CAC classics: cueing hierarchies and word retrieval: a therapy program. *Aphasiology* 19 77–92. 10.1080/02687030444000363

[B93] Loubinoux I. Kronenberg G. Endres M. Schumann-Bard P. Freret T. Filipkowski R. K. (2012). Post-stroke depression: mechanisms, translation and therapy. *J. Cell. Mol. Med.* 16 1961–1969. 10.1111/j.1582-4934.2012.01555.x 22348642 PMC3822966

[B94] Lytton W. W. Williams S. T. Sober S. J. (1999). Unmasking unmasked: neural dynamics following stroke. *Prog. Brain Res.* 121 203–218. 10.1016/S0079-6123(08)63075-7 10551028

[B95] Maher L. M. Kendall D. Swearengin J. A. Rodriguez A. Leon S. A. Pingel K. (2006). A pilot study of use-dependent learning in the context of constraint induced language therapy. *J. Int. Neuropsychol. Soc.* 12:843. 10.1017/S1355617706061029 17064447

[B96] Marangolo P. (2013). tDCS over the left inferior frontal cortex improves speech production in aphasia. *Front. Hum. Neurosci.* 7:539. 10.3389/fnhum.2013.00539 24046740 PMC3764371

[B97] Marangolo P. (2017). The potential effects of transcranial direct current stimulation (tDCS) on language functioning: combining neuromodulation and behavioral intervention in aphasia. *Neurosci. Lett.* 719:133329. 10.1016/j.neulet.2017.12.057 29289680

[B98] Marangolo P. Caltagirone C. (2014). Options to enhance recovery from aphasia by means of non-invasive brain stimulation and action observation therapy. *Expert Rev. Neurother.* 14 75–91. 10.1586/14737175.2014.864555 24308276

[B99] Marangolo P. Bonifazi S. Tomaiuolo F. Craighero L. Coccia M. Altoè G. (2010). Improving language without words: first evidence from aphasia. *Neuropsychologia* 48 3824–3833. 10.1016/j.neuropsychologia.2010.09.025 20887740

[B100] Marangolo P. Cipollari S. Fiori V. Razzano C. Caltagirone C. (2012). Walking but not barking improves verb recovery: implications for action observation treatment in aphasia rehabilitation. *PLoS One* 7:e38610. 10.1371/journal.pone.0038610 22719906 PMC3374821

[B101] Marangolo P. Fiori V. Caltagirone C. Pisano F. Priori A. (2018). Transcranial cerebellar direct current stimulation enhances verb generation but not verb naming in poststroke aphasia. *J. Cogn. Neurosci.* 30 188–199. 10.1162/jocn_a_01201 29064340

[B102] Marangolo P. Fiori V. Cipollari S. Campana S. Razzano C. Di Paola M. (2013a). Bihemispheric stimulation over left and right inferior frontal region enhances recovery from apraxia of speech in chronic aphasia. *Eur. J. Neurosci.* 38 3370–3377. 10.1111/ejn.12332 23930827

[B103] Marangolo P. Fiori V. Di Paola M. Cipollari S. Razzano C. Oliveri M. (2013b). Differential involvement of the left frontal and temporal regions in verb naming: a tDCS treatment study. *Restor. Neurol. Neurosci.* 31 63–72. 10.3233/RNN-120268 23142815

[B104] Marangolo P. Fiori V. Gelfo F. Shofany J. Razzano C. Caltagirone C. (2014b). Bihemispheric tDCS enhances language recovery but does not alter BDNF levels in chronic aphasic patients. *Restor. Neurol. Neurosci.* 32 367–379. 10.3233/RNN-130323 24398720

[B105] Marangolo P. Fiori V. Campana S. Calpagnano M. A. Razzano C. Caltagirone C. (2014a). Something to talk about: enhancement of linguistic cohesion through tdcs in chronic non fluent aphasia. *Neuropsychologia* 53 246–256. 10.1016/j.neuropsychologia.2013.12.003 24333381

[B106] Marangolo P. Fiori V. Sabatini U. De Pasquale G. Razzano C. Caltagirone C. (2016). Bilateral transcranial direct current stimulation language treatment enhances functional connectivity in the left hemisphere: preliminary data from aphasia. *J. Cogn. Neurosci.* 28 724–738. 10.1162/jocn_a_00927 26807842

[B107] Marangolo P. Fiori V. Shofany J. Gili T. Caltagirone C. Cucuzza G. (2017). Moving beyond the brain: transcutaneous spinal direct current stimulation in post-stroke aphasia. *Front. Neurol.* 8:400. 10.3389/fneur.2017.00400 28848492 PMC5550684

[B108] Marangolo P. Marinelli C. V. Bonifazi S. Fiori V. Ceravolo M. G. Provinciali L. (2011). Electrical stimulation over the left inferior frontal gyrus (IFG) determines long-term effects in the recovery of speech apraxia in three chronic aphasics. *Behav. Brain Res.* 225 498–504. 10.1016/j.bbr.2011.08.008 21856336

[B109] Maresca G. Maggio M. G. Latella D. Cannavò A. De Cola M. C. Portaro S. (2019). Toward Improving poststroke aphasia: a pilot study on the growing use of telerehabilitation for the continuity of care. *J. Stroke Cerebrovasc. Dis.* 28:104303. 10.1016/j.jstrokecerebrovasdis.2019.104303 31371144

[B110] Marini A. Galetto V. Tatu K. Duca S. Geminiani G. Sacco K. (2016). Recovering two languages with the right hemisphere. *Brain Lang.* 159 35–44. 10.1016/j.bandl.2016.05.014 27289209

[B111] Marshall R. S. Perera G. M. Lazar R. M. Krakauer J. W. Constantine R. C. DeLaPaz R. L. (2000). Evolution of cortical activation during recovery from corticospinal tract infarction. *Stroke* 31 656–661. 10.1161/01.STR.31.3.656 10700500

[B112] Meinzer M. Darkow R. Lindenberg R. Flöel A. (2016). Electrical stimulation of the motor cortex enhances treatment outcome in post-stroke aphasia. *Brain* 139 1152–1163. 10.1093/brain/aww002 26912641

[B113] Mineo L. Concerto C. Patel D. Mayorga T. Chusid E. Infortuna C. (2018). Modulation of sensorimotor circuits during retrieval of negative autobiographical memories: exploring the impact of personality dimensions. *Neuropsychologia* 110 190–196. 10.1016/j.neuropsychologia.2017.04.016 28404231

[B114] Miniussi C. Harris J. A. Ruzzoli M. (2013). Modelling noninvasive brain stimulation in cognitive neuroscience. *Neurosci. Biobehav. Rev.* 37 1702–1712. 10.1016/j.neubiorev.2013.06.014 23827785

[B115] Murase N. Duque J. Mazzocchio R. Cohen L. G. (2004). Influence of interhemispheric interactions on motor function in chronic stroke. *Ann. Neurol.* 55 400–409. 10.1002/ana.10848 14991818

[B116] Naro L. D. Spinello R. S. Cantone M. Iurato L. Mazzù I. D. Occhipinti C. (2021). Rehabilitative treatment in a case of aphasia as onset of multiple sclerosis. *Neurol. Sci.* 42 3919–3921. 34125324 10.1007/s10072-021-05364-2

[B117] Nitsche M. A. Doemkes S. Karakose T. Antal A. Liebetanz D. Lang N. (2007). Shaping the effects of transcranial direct current stimulation of the human motor cortex. *J. Neurophysiol.* 97 3109–3117. 10.1152/jn.01312.2006 17251360

[B118] Nitsche M. A. Fricke K. Henschke U. Schlitterlau A. Liebetanz D. Lang N. (2003). Pharmacological modulation of cortical excitability shifts induced by transcranial direct current stimulation in humans. *J. Physiol.* 553 293–301. 10.1113/jphysiol.2003.049916 12949224 PMC2343495

[B119] Norise C. Sacchetti D. Hamilton R. (2017). Transcranial direct current stimulation in post-stroke chronic aphasia: the impact of baseline severity and task specificity in a pilot sample. *Front. Hum. Neurosci.* 11:260. 10.3389/fnhum.2017.00260 28611609 PMC5447043

[B120] O’Connell N. E. Cossar J. Marston L. Wand B. M. Bunce D. Moseley G. L. (2012). Rethinking clinical trials of transcranial direct current stimulation: participant and assessor blinding is inadequate at intensities of 2mA. *PLoS One* 7:e47514. 10.1371/journal.pone.0047514 23082174 PMC3474749

[B121] Özdemir E. Norton A. Schlaug G. (2006). Shared and distinct neural correlates of singing and speaking. *Neuroimage* 33 628–635. 10.1016/j.neuroimage.2006.07.013 16956772

[B122] Pani E. Zheng X. Wang J. Norton A. Schlaug G. (2016). Right hemisphere structures predict poststroke speech fluency. *Neurology* 86 1574–1581. 10.1212/WNL.0000000000002613 27029627 PMC4844242

[B123] Pascual-Leone A. Amedi A. Fregni F. Merabet L. B. (2005). The plastic human brain cortex. *Annu. Rev. Neurosci.* 28 377–401. 10.1146/annurev.neuro.27.070203.144216 16022601

[B124] Pestalozzi M. I. Di Pietro M. Martins Gaytanidis C. Spierer L. Schnider A. Chouiter L. (2018). Effects of prefrontal transcranial direct current stimulation on lexical access in chronic poststroke aphasia. *Neurorehabil. Neural Repair* 32 913–923. 10.1177/1545968318801551 30269644

[B125] Pisano F. Caltagirone C. Incoccia C. Marangolo P. (2021). DUAL-tDCS treatment over the temporo-parietal cortex enhances writing skills: first evidence from chronic post-stroke aphasia. *Life* 11:343. 10.3390/life11040343 33919714 PMC8070712

[B126] Pulvermüller F. Neininger B. Elbert T. Mohr B. Rockstroh B. Koebbel P. (2001). Constraint-induced therapy of chronic aphasia after stroke. *Stroke* 32 1621–1626. 10.1161/01.STR.32.7.1621 11441210

[B127] Racette A. Bard C. Peretz I. (2006). Making non-fluent aphasics speak: sing along! *Brain* 129 2571–2584. 10.1093/brain/awl250 16959816

[B128] Richards K. Singletary F. Koehler S. Crosson B. Rothi L. J. G. (2002). Treatment of nonfluent aphasia through the pairing of a non-symbolic movement sequence and naming. *J. Rehabil. Res. Dev.* 39 7–16. 17638142

[B129] Richardson J. Datta A. Dmochowski J. Parra L. C. Fridriksson J. (2015). Feasibility of using high-definition transcranial direct current stimulation (HD-tDCS) to enhance treatment outcomes in persons with aphasia. *NeuroRehabilitation* 36 115–126. 10.3233/NRE-141199 25547776 PMC5764169

[B130] Rodriguez A. D. Raymer A. M. Gonzalez L. J. (2006). Effects of gesture + verbal and semantic-phonologic treatments for verb retrieval in aphasia. *Aphasiology* 20 286–297. 10.1080/02687030500474898

[B131] Rose M. L. (2013). Releasing the constraints on aphasia therapy: the positive impact of gesture and multimodality treatments. *Am. J. Speech Lang. Pathol.* 22 S227–S239. 10.1044/1058-0360(2012/12-0091) 23695899

[B132] Rosso C. Arbizu C. Dhennain C. Lamy J. C. Samson Y. (2018). Repetitive sessions of tDCS to improve naming in post-stroke aphasia: insights from an individual patient data (IPD) meta-analysis. *Restor. Neurol. Neurosci.* 36 107–116. 29439369 10.3233/RNN-170783

[B133] Rosso C. Perlbarg V. Valabregue R. Arbizu C. Ferrieux S. Alshawan B. (2014). Broca’s area damage is necessary but not sufficient to induce after-effects of cathodal tDCS on the unaffected hemisphere in post-stroke aphasia. *Brain Stimul.* 7 627–635. 10.1016/j.brs.2014.06.004 25022472

[B134] Saidmanesh M. Hamid R. P. Abdollah A. Reza N. Hamed E. (2012). Effects of transcranial direct current stimulation on working memory in patients with non fluent aphasia disorder. *Res. J. Biol. Sci.* 7 290–296. 10.3923/rjbsci.2012.290.296

[B135] Sandars M. Cloutman L. Woollams A. M. (2016). Taking sides: an integrative review of the impact of laterality and polarity on efficacy of therapeutic transcranial direct current stimulation for anomia in chronic poststroke aphasia. *Neural Plast.* 2016 8428256. 10.1155/2016/8428256 26819777 PMC4706968

[B136] Saur D. Lange R. Baumgaertner A. Schraknepper V. Willmes K. Rijntjes M. (2006). Dynamics of language reorganization after stroke. *Brain* 129 1371–1384. 10.1093/brain/awl090 16638796

[B137] Schlaug G. Marchina S. Wan C. Y. (2011). The use of non-invasive brain stimulation techniques to facilitate recovery from post-stroke aphasia. *Neuropsychol. Rev.* 21:288. 10.1007/s11065-011-9181-y 21842404 PMC3176334

[B138] Schuell H. Jenkins J. J. Jiminez-Pabon E. (1964). *Aphasia in Adults.* New York, NY: Harper Medical Division.

[B139] Shah-Basak P. P. Norise C. Garcia G. Torres J. Faseyitan O. Hamilton R. H. (2015). Individualized treatment with transcranial direct current stimulation in patients with chronic non-fluent aphasia due to stroke. *Front. Hum. Neurosci.* 9:201. 10.3389/fnhum.2015.00201 25954178 PMC4404833

[B140] Shah-Basak P. P. Wurzman R. Purcell J. B. Gervits F. Hamilton R. (2016). Fields or flows? A comparative metaanalysis of transcranial magnetic and direct current stimulation to treat post-stroke aphasia. *Restor. Neurol. Neurosci.* 34 537–558. 10.3233/RNN-150616 27163249

[B141] Simmons-Mackie N. Worrall L. E. Frattali C. M. (2000). “Social approaches to the management of aphasia,” in *Neurogenic Communication Disorders: A Functional Approach*, Vol. 10 eds Worrall L. Frattal C. (New York, NY: Thieme), 172–173.

[B142] Simonetta-Moreau M. (2014). Non-invasive brain stimulation (NIBS) and motor recovery after stroke. *Ann. Phys. Rehabil. Med.* 57 530–542. 10.1016/j.rehab.2014.08.003 25193774

[B143] Small S. L. Llano D. A. (2009). Biological approaches to aphasia treatment. *Curr. Neurol. Neurosci. Rep.* 9 443–450. 10.1007/s11910-009-0066-x 19818231 PMC3405725

[B144] Sparks R. W. (2008). “Melodic intonation therapy,” in *Language Intervention Strategies in Aphasia and Related Neurogenic Communication Disorders*, ed. Chapey R. (Baltimore, MD: Lippincott, Williams & Wilkins), 837–851.

[B145] Spielmann K. Van de Sandt-Koenderman W. M. E. Heijenbrok-Kal M. H. Ribbers G. M. (2018). Comparison of two configurations of transcranial direct current stimulation for treatment of aphasia. *J. Rehabil. Med.* 50 527–533. 10.2340/16501977-2338 29736552

[B146] Stagg C. J. Nitsche M. A. (2011). Physiological basis of transcranial direct current stimulation. *Neuroscientist* 17 37–53. 10.1177/1073858410386614 21343407

[B147] Stagg C. J. Best J. G. Stephenson M. C. O’Shea J. Wylezinska M. Kincses Z. T. (2009). Polarity-sensitive modulation of cortical neurotransmitters by transcranial stimulation. *J. Neurosci.* 29 5202–5206. 10.1523/JNEUROSCI.4432-08.2009 19386916 PMC6665468

[B148] Stahl B. Darkow R. von Podewils V. Meinzer M. Grittner U. Reinhold T. (2019). Transcranial direct current stimulation to enhance training effectiveness in chronic post-stroke aphasia: a randomized controlled trial protocol. *Front. Neurol.* 10:1089. 10.3389/fneur.2019.01089 31695667 PMC6817733

[B149] Theodoros D. Hill A. Russell T. Ward E. Wootton R. (2008). Assessing acquired language disorders in adults via the Internet. *Telemed. eHealth* 14 552–559. 10.1089/tmj.2007.0091 18729754

[B150] Thompson C. K. den Ouden D. B. (2008). Neuroimaging and recovery of language in aphasia. *Curr. Neurol. Neurosci. Rep.* 8 475–483. 10.1007/s11910-008-0076-0 18957184 PMC3079407

[B151] Ulanov M. A. Shtyrov Y. Y. Stroganova T. A. (2019). Transcranial direct current stimulation as a tool to induce language recovery in patients with post-stroke aphasia. *Neurosci. Behav. Physiol.* 49 1169–1180. 10.1007/s11055-019-00854-5

[B152] Van de Sandt-Koenderman W. M. E. (2011). Aphasia rehabilitation and the role of computer technology: can we keep up with modern times? *Int. J. Speech Lang. Pathol.* 13 21–27. 10.3109/17549507.2010.502973 21329407

[B153] Vargha-Khadem F. Carr L. J. Isaacs E. Brett E. Adams C. Mishkin M. (1997). Onset of speech after left hemispherectomy in a nine-year-old boy. *Brain* 120 159–182. 10.1093/brain/120.1.159 9055805

[B154] Vestito L. Rosellini S. Mantero M. Bandini F. (2014). Long-term effects of transcranial direct-current stimulation in chronic post-stroke aphasia: a pilot study. *Front. Hum. Neurosci.* 8:785. 10.3389/fnhum.2014.00785 25352798 PMC4196539

[B155] Vines B. W. Norton A. C. Schlaug G. (2009). “Stimulating music: combining melodic intonation therapy with transcranial DC stimulation to facilitate speech recovery after stroke,” in *Transmitters and Modulators in Health and Disease*, eds Shioda S. Homma I. Kato N. (Tokyo: Springer), 103–114. 10.1007/978-4-431-99039-0_8

[B156] Vines B. W. Norton A. C. Schlaug G. (2011). Non-invasive brain stimulation enhances the effects of melodic intonation therapy. *Front. Psychol.* 2:230. 10.3389/fpsyg.2011.00230 21980313 PMC3180169

[B157] Watila M. M. Balarabe S. A. (2015). Factors predicting post-stroke aphasia recovery. *J. Neurol. Sci.* 352 12–18. 10.1016/j.jns.2015.03.020 25888529

[B158] Wepman J. M. (1972). Aphasia therapy: a new look. *J. Speech Hear. Disord.* 37 203–214. 10.1044/jshd.3702.203 4337451

[B159] Woodhead Z. V. Kerry S. J. Aguilar O. M. Ong Y. H. Hogan J. S. Pappa K. (2018). Randomized trial of iReadMore word reading training and brain stimulation in central alexia. *Brain* 141 2127–2141. 10.1093/brain/awy138 29912350 PMC6118228

[B160] Zeiler S. R. (2019). Should we care about early post-stroke rehabilitation? Not yet, but soon. *Curr. Neurol. Neurosci. Rep.* 19:13. 10.1007/s11910-019-0927-x 30788609

[B161] Zettin M. Leopizzi M. Galetto V. (2018). How does language change after an intensive treatment on imitation? *Neuropsychol. Rehabil.* 29 1332–1358. 10.1080/09602011.2017.1406861 29322866

[B162] Zhao Q. Wang J. Li Z. Song L. Li X. (2021). Effect of anodic transcranial direct current stimulation combined with speech language therapy on nonfluent poststroke aphasia. *Neuromodulation* 24 923–929. 10.1111/ner.13337 33624330

