# Supplementary material for: Transcranial Direct-Current Stimulation and Behavioral Training, a Promising Tool for a Tailor-Made Post-stroke Aphasia Rehabilitation: A Review
Source: Front Hum Neurosci. 2021 Dec 20;15:742136. doi: 10.3389/fnhum.2021.742136 (PMC8722401; doi:10.3389/fnhum.2021.742136)
Supplement: Supplementary file 1 [file Table_1.pdf]

Supplementary Table 1: Experimental Studies

Author(s)	Year	Sample	tDCS montage	Stimulation site	Intensity	Duration of tDCS stimulation	Number of tDCS sessions	Duration of treatment	Paired task
Kang et al.	2007	5 patients with chronic aphasia	C-tDCS	Broca's homologous areas of the right hemisphere	2mA	20 min	5	5 days	Speech Therapy
Vines et al.	2009	6 patients with chronic aphasia	A-tDCS	Right IFG	1.2 mA	20 min	3 per condition	6 days	Melodic Intonation Therapy
Baker et al.	2010	10 patients with chronic aphasia	A-tDCS	Left perilesional areas	1 mA	20 min	5 active, 5 sham	5 days + 1 week interval between conditions	Picture-Word Matching Task
Fiori et al.	2011	3 patients with chronic aphasia and 10 healthy subjects	A-tDCS	Wernicke's Area	1 mA	20 min	5 active, 5 sham	2 weeks + 1 week interval between conditions	Picture Naming Task
Floël et al.	2011	12 patients with chronic aphasia	A-tDCS or C-tDCS	Right temporo-parietal areas	1 mA	20 min	3 (1 active anodal, 1 active cathodal, 1 sham. Randomized.)	3 days + 3 week interval between conditions	Anomia Training
Fridriksson et al.	2011	8 patients with chronic aphasia	A-tDCS	Perilesional areas	1 mA	20 min	5 active, 5 sham	2 weeks + 1 week interval between conditions	Computerised Anomia Treatment
Jung et al.	2011	37 patients with chronic aphasia	C-tDCS	Broca's homologous areas of the right hemisphere	1 mA	30 min	10	2 to 3 weeks	Speech and Language Therapy
Kang et al.	2011	10 patients with chronic aphasia	C-tDCS	Broca's homologous areas of the right hemisphere	2 mA	20 min	5 active, 5 sham	2 weeks + 1 week interval between conditions	Word Retrieval Training
Marangolo et al.	2011	3 patients with chronic aphasia	A-tDCS	Broca's Area	1 mA	20 min	5 active, 5 sham	2 weeks + 6 days interval between conditions	Word Repetition Task
Vines et al.	2011	6 patients with chronic aphasia	A-tDCS	Broca's homologous areas of the right hemisphere	1.2 mA	20 min	3 active, 3 sham	6 days + 1 week interval between conditions	Melodic Intonation Therapy
Saidmanesh et al.	2012	20 patients with chronic aphasia	A-tDCS	Left prefrontal dorsolateral areas	2 mA	20 min	10 active, 10 sham	20 days + 1 week interval between conditions	Verbal fluency and working memory tasks
Fiori et al.	2013	7 patients with chronic aphasia	A-tDCS	Left frontal and temporal areas	1 mA	20 min	5 for each condition, 5 sham	15 days + 6 days interval between conditions	Picture-Naming Task
Lee et al.	2013	11 patients with chronic aphasia	A-tDCS or bihemispheric tDCS	Two pairs of electrodes employed: (1) anode on Broca's area and reference on left buccinator muscle; (2) cathode on Broca's right homologous areas and reference on right buccinator muscle	2 mA	30 min	2	2 days + 1 day interval between conditions	Speech and Language Therapy
Marangolo et al.	2013	7 patients with chronic aphasia	A-tDCS	Broca's or Wernicke's Areas	1 mA	20 min	5 per condition	15 days + 6 days interval between conditions	Naming Task
Marangolo et al.	2013	12 patients with chronic aphasia	A-tDCS	Broca's Area	1 mA	20 min	5 active, 5 sham	2 weeks + 14 days interval between conditions	Conversational Therapy
Marangolo et al.	2013	8 patients with chronic aphasia	Bihemispheric stimulation	Anode on Broca's area, cathode on right homologous areas	2 mA	20 min	10 active, 10 sham	20 days + 14 days interval between sessions	Language Therapy for apraxia

Marangolo et al.	2014	7 patients with chronic aphasia	Bihemispheric stimulation	Anode on perilesional areas, cathode on homologous areas of the right hemisphere	2 mA	20 min	10 active, 10 sham	20 days + 14 days interval between sessions	Speech and Language Therapy
Marangolo et al.	2014	8 patients with chronic aphasia and 20 healthy subjects	A-tDCS	Broca's or Wernicke's Areas	1 mA	20 min	20 active, 10 sham	30 days + 14 days interval between sessions	Conversational Therapy
Rosso et al.	2014	25 patients with chronic aphasia	C-tDCS	Left personalised montage based on the lesion site	1 mA	15 min	1 active, 1 sham	1 day + 2 hours interval between sessions	Picture Naming Task
Vestito et al.	2014	3 patients with chronic aphasia	A-tDCS	Perilesional areas	1.5 mA	20 min	5 active, 5 sham	5 days (both conditions every day) + 2 days interval between sessions	Naming Task
Campana et al.	2015	20 patients with chronic aphasia	A-tDCS	Broca's Area	2 mA	20 min	5 active, 5 sham	1 week + 14 days interval between conditions	Conversational Therapy
Cipollari et al.	2015	6 patients with chronic aphasia	A-tDCS o C-tDCS	Broca's homologous areas of the right hemisphere	2 mA	20 min	15 active per condition	3 weeks + 14 days interval between conditions	Melodic IntonationTherapy
De Aguiar et al.	2015	9 patients with chronic aphasia	Bihemispheric tDCS	Anode on perilesional areas, cathode on homologous areas of the right hemisphere	1 mA	20 min	10 active, 10 sham	10 weeks	SLT based on ACTION treatment
Richardson et al.	2015	8 patients with chronic aphasia	A-tDCS (conventional and HD-tDCS)	Personalised montage on left frontal lobe	1 mA	20 min	5 per condition	5 weeks	Computerised Anomia Treatment
Shah-Basak et al.	2015	1st phase: 12 patients with chronic aphasia; 2nd phase: 5 patients	A-tDCS or C-tDCS	Frontal personalised montage	2 mA	20 min	1st phase: 4<br>2nd phase: 10	2 weeks per condition	Picture Naming Task
Basat et al.	2016	7 patients with chronic aphasia	A-tDCS or C-tDCS	Broca's or Wernicke's Areas	2 mA	10 min	6 active, 6 sham	2 weeks	Psycholinguistic Assessment of Language Processing in Aphasia (PALPA), Written Word Association test, Picture Association test
Marangolo et al.	2016	9 patients with chronic aphasia	Bihemispheric stimulation	Anode on Broca's Area, cathode on right homologous areas	2 mA	20 min	5 per condition	3 weeks	Speech and Language Therapy
Meinzer et al.	2016	26 patients with chronic aphasia	A-tDCS	Left motor cortex	1 mA	20 min	8 per group	2 weeks	Computer-assisted Naming Treatment
Branscheidt et al.	2017	16 patients with chronic aphasia	A-tDCS	Left primary motor cortex	2 mA	20 min	1 active, 1 sham	2 days + 7 days interval between conditions	Lexical Decision Task
Darkow et al.	2017	16 patients with chronic aphasia	A-tDCS	Left primary motor cortex	1 mA	20 min	1 active, 1 sham	2 days + 1 week interval between conditions	Picture-object naming of familiar terms
Marangolo et al.	2017	14 patients with chronic aphasia	A-tsDCS	10th thoracic vertebra	2 mA	20 min	5 per condition	15 days + 6 days interval in between conditions	Verb and Noun Naming Task
Norise et al.	2017	9 patients with chronic aphasia	A-tDCS or C-tDCS	Left or right frontal areas	2 mA	20 min	1st phase: 1 per condition;<br>2nd phase: 10 per group	2 months	Picture Naming Task
Cramer	2018	74 patients	A-tDCS	Broca's Area	1 mA	45 min	15 per group	3 weeks	Speech Therapy
Fridriksson et al.	2018	74 patients with chronic aphasia	A-tDCS	Perilesional areas	1 mA	20 min	15 per group	3 weeks	Computerised Anomia Treatment
Fridriksson et al.	2018	74 patients with chronic aphasia	A-tDCS	Left temporoparietal areas	1 mA	20 min	15 per group	3 weeks	Picture-Word Matching Task
Marangolo et al.	2018	12 patients with chronic aphasia	C-tDCS	Right cerebellar hemisphere	2 mA	20 min	5 per condition	3 weeks	Language Treatment for Verb Improvement

Pestalozzi et al.	2018	14 patients with chronic aphasia	A-tDCS	Left prefrontal dorsolateral areas	1 mA	20 min	1 active, 1 sham	2 days + 1 week interval between conditions	Picture Naming Task and repetition
Spielmann et al.	2018	13 patients with chronic aphasia	A-tDCS	Broca's Area or left temporal areas	1 mA	20 min	2 active, 1 sham	4 weeks	Word-Finding Therapy
Woodhead et al.	2018	21 patients with chronic aphasia and alexia	A-tDCS	Perilesional areas	2 mA	20 min	11 per condition	4 months	iReadMore
Fiori et al.	2019	20 patients with chronic aphasia	C-HD-tDCS	Broca's homologous areas of the right hemisphere	Group 1 1mA; Group 2 2mA	20 min	5 per condition	2 weeks + 1 week interval between condition	Verb Naming Task
Stahl et al.	2019	26 patients with chronic aphasia	A-tDCS	Left motor cortex	1 mA	20 min	12.5 hours weekly (2 daily sessions)	3 weeks	Intensive Speech-Language Therapy
Guillouët et al.	2020	10 patients with chronic aphasia	Bihemispheric tDCS	Anode on Broca's area; cathode on homologous areas of the right hemisphere	2 mA	20 min	15 per condition	3 weeks per condition + 1 week interval between conditions	Speech and Language Therapy
Ihara et al.	2020	6 patients with chronic aphasia	A-tDCS	Anode on Broca's area; cathode on homologous areas of the right hemisphere	1.5 mA	20 min	4 per condition	4 days + 3 weeks interval between conditions	Word-Picture Matching Task
Cherney et al.	2021	12 patients with chronic aphasia	A-tDCS or C-tDCS	Perilesional areas	1 mA	13 min	30	6 weeks	Speech and Language Therapy
Pisano et al	2021	14 patients with chronic aphasia	Bihemispheric tDCS	Temporoparietal areas	2 mA	20 min	10	2 weeks	Writing Tasks
Zhao et al.	2021	18 patients with chronic aphasia	A-tDCS	Left inferior frontal gyrus	2 mA	20 min	20	4 weeks	Speech and Language Therapy
